# Alkyl Chain Length Governs Structure, Conformation and Antimicrobial Activity in Poly(alkylene biguanide)

**DOI:** 10.3390/polym18030390

**Published:** 2026-02-01

**Authors:** Enas Al-Ani, Khalid Doudin, Andrew J. McBain, Zeeshan Ahmad, Sally Freeman

**Affiliations:** 1 Division of Pharmacy and Optometry, University of Manchester, Manchester M13 9PL, UK; 2 Department of Mathematical and Physical Sciences, The University of Sheffield, Sheffield S10 2TN, UK

**Keywords:** poly(alkylene biguanide), polymeric biguanides, cationic PHMB, antimicrobial structure–activity relationship, polydispersity, hydrodynamic diameter, DOSY NMR, invers laplace transform

## Abstract

Poly(hexamethylene biguanide) (PHMB) is a polycationic antimicrobial polymer exhibiting broad-spectrum activity against bacteria, fungi, and viruses, and is widely used in medical settings for infection prevention and control. However, the relationship between chemical structure and antimicrobial activity remains unclear. In this study, we synthesised and characterised a series of polymeric biguanides with systematically varied alkyl chain lengths to examine the effects of structural variation on physicochemical properties and antimicrobial activity. H NMR spectroscopy and FTIR confirmed successful polymerisation. Solubility measurements revealed a progressive decrease in aqueous solubility with increasing alkyl chain length, consistent with increased hydrophobicity. Dynamic light scattering indicated reversible folding and unfolding of polymer chains in aqueous solution, with stabilisation at higher concentrations. Diffusion-ordered spectroscopy was used to calculate hydrodynamic diameters and polydispersity indices. Antimicrobial assays against *Staphylococcus aureus* and *Pseudomonas aeruginosa* showed that polymers containing heptamethylene and octamethylene chains exhibited the highest antibacterial activity, whereas tetramethylene- and pentamethylene-containing polymers showed greater fungicidal activity against *Candida albicans*. Highly hydrophobic polymers showed increased aggregation, resulting in reduced antimicrobial efficacy. Overall, these results indicate that both charge density and alkyl chain length are key determinants of antimicrobial activity. This polymeric biguanide series provides a platform for further investigation of structure–activity relationships and mechanisms of action against pathogenic microorganisms and their biofilms.

## 1. Introduction

The World Health Organisation (WHO) has identified antimicrobial resistance (AMR) as one of the top 10 global health threats [1]. AMR contributes to approximately 5 million deaths annually worldwide, and the estimated additional healthcare costs could reach USD1 trillion by 2050 [2]. This is attributed to the growing prevalence of pathogens resistant to currently available antimicrobials. As a result, antiseptics are increasingly being used as alternatives, particularly in the treatment of topical infections [3,4]. Antiseptic biguanides are potent antimicrobials with broad-spectrum activity against bacteria, fungi, viruses and protozoa [5,6]. The most commonly used biguanides in medical products for their antimicrobial activities are chlorhexidine and polyhexamethylene biguanide (PHMB, also known as polyhexanide or polyaminopropyl biguanide, according to the International Nomenclature of Cosmetic Ingredients (INCI) [7]). Compared with PHMB, chlorhexidine is associated with a higher incidence of allergic reactions [8].

PHMB is one of the most extensively studied and widely used biguanides. It was first synthesised by Rose and Swain in 1953 [9], followed by subsequent studies investigating its synthesis and properties [10,11]. PHMB is a polymer widely recognised for its antimicrobial efficacy, chemical stability, and relatively low toxicity, making it suitable for a range of medical and cosmetic applications [12]. It is currently used as a disinfectant and antiseptic in wound care products and contact lens solutions, and in bladder irrigation solutions and certain ophthalmic formulations as a preservative [12,13,14]. More recently, PHMB has been licenced for the treatment of Acanthamoeba keratitis [15]. In addition, PHMB has shown promising potential in gene and drug delivery applications [16]. Although PHMB has been explored as a surface coating for medical devices, its use in implantable biomedical devices remains limited and largely investigational [17,18]. Despite its strong antimicrobial potency, cases of treatment failure or incomplete eradication of infection have been reported [19,20]. The antimicrobial activity of PHMB is proposed to result from the formation of salt bridges between its cationic biguanide groups and anionic phospholipids in the cell membrane [21,22]. This interaction is reportedly followed by the insertion of the hydrophobic hexamethylene segments into the nonpolar lipid bilayer, facilitating membrane disruption or enabling PHMB translocation into the cell [21,22]. However, the impact of the hydrocarbon chain length has not been determined, which may be attributed to the lack of availability of other polymers that are structurally similar to PHMB.

Although the antimicrobial activity of PHMB has been widely attributed to electrostatic interactions between its cationic biguanide groups and anionic membrane components, followed by insertion of its hydrophobic hexamethylene segments into lipid bilayers, the extent to which these mechanisms depend on polymer architecture remains poorly defined. In particular, the role of hydrocarbon chain length in governing polymer conformation, aggregation behaviour, and antimicrobial efficacy has not been systematically investigated. This limitation largely reflects the lack of structurally comparable polymeric biguanides beyond PHMB itself. As a result, current mechanistic models implicitly assume that PHMB represents an optimal or generalisable structure, despite accumulating evidence that treatment failure, incomplete microbial eradication, and organism-specific responses can occur.

To address this gap, we synthesised and characterised a homologous series of Poly(alkylene biguanide) with systematically varied alkyl chain lengths (C4–C10). By decoupling charge density from hydrophobic segment length, this series enables direct interrogation of how polymer structure influences physicochemical behaviour, solution conformation, and antimicrobial activity against representative Gram-positive, Gram-negative, and fungal pathogens. This structure–activity framework provides new mechanistic insight into polymeric biguanide antimicrobial function and establishes design principles for optimising efficacy while minimising aggregation-driven loss of activity.

## 2. Materials and Methods

### 2.1. Materials

Poly(hexamethylene biguanide) hydrochloride (PHMB) 98% was obtained from Cambridge Bioscience, UK. Sodium dicyanamide 97% was obtained from Biosynth (Carbosynt), UK. *Staphylococcus aureus* subsp. aureus WDCM 00,032 Vitroids™ (ATCC^®^ 6538) was obtained from Merck, UK. *Pseudomonas aeruginosa* WDCM 00,026 Vitroids™ (ATCC^®^ 9027) and *Candida albicans* WDCM 00,054 Vitroids™ (ATCC^®^ 10231) were obtained from Scientific Laboratories Supplies, UK. Tryptic soy broth (TSB), Tryptic soy agar (TSA) and Sabauroud dextrose agar (SDA) were obtained from Formedium Ltd., UK. Diaminobutane dihydrochloride 99%, diaminopentane dihydrochloride 98%, diaminohexane dihydrochloride 99%, diaminoheptane 98%, 1,9-diaminononane 98% and Sabauroud dextrose broth (SDB) were obtained from Merck, UK. Diaminooctane >98% and diaminodecane >98% were obtained from TCI, Belgium. Ultrapure water (resistivity 18.2 MΩ·cm, obtained from a Milli-Q purification system equipped with a UV lamp) was used in all experiments.

### 2.2. Methods

**Figure 1:**
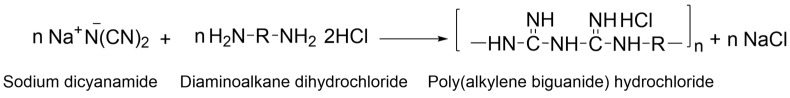
Synthesis of Poly(alkylene biguanide).

**Figure 2:**
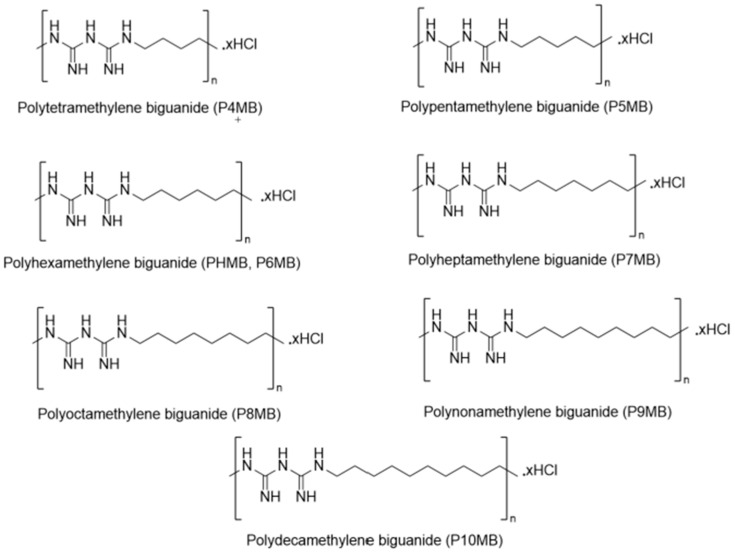
Chemical structures of the synthesised Poly(alkylene biguanide).

A range of Poly(alkylene biguanide) was synthesised using a polycondensation method (Figure 1) [9,10]. The present study focuses on the structural and physicochemical characterisation of these compounds. The synthesised polymers included polytetramethylene biguanide hydrochloride (P4MB), polypentamethylene biguanide hydrochloride (P5MB), polyhexamethylene biguanide hydrochloride (P6MB), polyheptamethylene biguanide hydrochloride (P7MB), polyoctamethylene biguanide hydrochloride (P8MB), polynonamethylene biguanide hydrochloride (P9MB), and polydecamethylene biguanide hydrochloride (P10MB) (Figure 2).

### 2.3. UV/VIS Absorption

Test compounds were dissolved in distilled water and scanned using a PerkinElmer Lambda 25 UV/VIS spectrophotometer (PerkinElmer, Waltham, MA, USA). Spectra were recorded over the range of 200–400 nm using quartz cuvettes with a 1 cm path length at ambient temperature.

### 2.4. Solubility

Thermodynamic solubility was investigated by adding an excess of each polymer to ultrapure water. The polymer mixtures were then allowed to reach equilibrium at room temperature (21.0 ± 0.1 °C) in an orbital shaker set at 150 rpm for 24 h. After incubation, the samples were filtered and analysed using UV/Vis spectroscopy at an appropriate wavelength.

### 2.5. Calculated LogP

The logarithm of the partition coefficients (Log*P*) was calculated using ChemDraw (version 23.1.2).

### 2.6. Fourier-Transform Infrared Spectroscopy (FTIR)

FTIR spectra of the Poly(alkylene biguanide) were obtained using a Bruker Alpha II spectrometer (Bruker Optics, Ettlingen, Germany) equipped with an attenuated total reflectance (ATR) accessory. The powder samples were placed directly onto the diamond crystal, and uniform pressure was applied to ensure good contact between the sample and the crystal surface. Spectra were recorded in the range 4000–400 cm^−1^ with 128 scans per sample. Data acquisition and processing were performed using OPUS software.

### 2.7. Dynamic Light Scattering (DLS)

Volume distribution of the Poly(alkylene biguanide) was measured using a Malvern Zetasizer Nano ZS series instrument (Malvern Instruments, Malvern, UK) operating at 633 nm with a detection angle of 173° in backscatter mode. Ultrapure water was filtered through a 0.22 µm membrane filter, and polymer samples were filtered through 0.45 µm filters prior to analysis. Each sample was measured in triplicate under automatic settings at 25 °C. The refractive index of the polymer samples was estimated using machine-learning algorithms implemented via Docker software [23].

### 2.8. Nuclear Magnetic Resonance Spectroscopy (NMR)

^1^H NMR spectra of the Poly(alkylene biguanide) were recorded on a Bruker Avance III 400 MHz spectrometer at 298 K in D_2_O and DMSO-d_6_. Chemical shifts were referenced to residual solvent peaks (δ 4.7 ppm for HDO, δ 2.50 ppm for DMSO-d_5_).

### 2.9. Determination of the Molecular Weight of the Polymers Using End-Group Analysis

Molecular weight of the Poly(alkylene biguanide) was determined by end-group analysis using ^1^H NMR spectroscopy. Polymers were dissolved in deuterated solvents (DMSO-d_6_ or D_2_O), and spectra were recorded on a 400 MHz Bruker instrument. The degree of polymerisation (DP) was calculated from the integral values of the backbone methylene signals (at 1.1–1.7 ppm) and the integral values of the end-group methylene signal at ~2.75 ppm in DMSO-d_6_ (~2.9 ppm in D_2_O), which corresponds to the terminal –CH_2_–NH_2_ groups at both ends of the polymer chain. This approach assumes that polymer chains terminate with diamine-derived and dicyanamide-derived biguanide groups, consistent with the synthetic route and the most probable termination mechanism. Molecular weight was then obtained by multiplying the number of repeat units and end groups by their respective molecular masses. The chosen end groups provide the most chemically probable estimation for polymers prepared by this method.

### 2.10. Diffusion-Ordered Spectroscopy (DOSY)

DOSY NMRs of the Poly(alkylene biguanide) were conducted to investigate the fractional hydrodynamic diameter and the polydispersity index. The experiments were performed on a Bruker Avance III 400 MHz spectrometer at 308 K using the ledbpgp2s pulse sequence. Spectra were recorded in D_2_O and DMSO-d_6_. Sixteen gradient steps were applied linearly from 2% to 95% of the maximum gradient strength. The diffusion delay (Δ) was 39.9 ms, and the gradient pulse duration (δ) was 4.8 ms for D_2_O and 6.4 ms for DMSO-d6. Data were processed using Bruker Dynamics Centre (v. 2.8.8), and diffusion coefficients were obtained by fitting signal attenuation to the Stejskal–Tanner equation.

Two-dimensional DOSY maps were generated using the Inverse Laplace Transform (ILT) algorithm with automatic regularisation and logarithmic diffusion grids ranging from 1 × 10^−12^ to 1 × 10^−9^ m^2^ s^−1^. The fractional hydrodynamic diameters corresponding to each diffusion component were calculated from the measured diffusion coefficients using the Stokes–Einstein equation, taking into account solvent viscosity at the experimental temperature (0.87 mPa.s for D_2_O and 1.628 mPa.s for DMSO-d_6_) [24,25]. The fractional percentages were determined using OriginPro 2025 (10.2.0.188) software by integrating the area under the diffusion distribution curves obtained from the ILT analysis.

The polydispersity index (PDI) was calculated elsewhere using the barycentric approach [26], based on the diffusion coefficients of the end-group (extremities) and the polymer backbone. ILT was implemented to determine the peak frequencies using the following equations:

PDI is obtained as the ratio of the mass average molar mass (*M_w_*) to the number-average molar mass (*M_n_*).

$$PDI=\frac{M_{w}}{M_{n}}$$

$$M_{n}=\frac{\sum niMi}{\sum ni}=NM$$

$$M_{w}=\frac{\sum miMi}{\sum mi}$$

where *ni* is the number of molecules of mass *Mi*, mi the corresponding total mass, and *N* the average chain length. Since the molecular weight is directly proportional to the diffusion coefficient, *D* can be used instead of *M*, and the appropriate fractal factor can be applied.

$$M\propto D^{-df}$$

$$PDI={\left(\frac{\langle D_{w}\rangle }{\langle D_{n}\rangle }\right)}^{-df}$$

where *D_w_* and *D_n_* are the mean diffusion coefficients obtained from the ILT analysis for the polymer backbone and the chain extremities (end-groups), respectively.

$$D_{n}=\frac{\sum Dn{\times A}_{ex}\left(Dn\right)}{\sum A_{ex}\left(Dn\right)}$$

$$D_{w}=\frac{\sum Dn{\times A}_{c}\left(Dn\right)}{\sum A_{c}\left(Dn\right)}$$

*A_ex_* and *A_c_* are the individual ILT intensities corresponding to the chain extremities and the polymer backbone, respectively.

To obtain the fractional hydrodynamic diameters using the barycentric approach, A_c_ was plotted against the diffusion coefficient (*D*). The regions of interest (ROIs), corresponding to the peaks for each fraction, were then manually selected. The diffusion coefficients within each ROI (*D_ROI_*) values were used to calculate the hydrodynamic diameters using the Stokes–Einstein equation [27].

$$D_{ROI}=\frac{KT}{6\pi \eta R_{H}}$$

where *K* is the Boltzmann constant, *T* is the absolute temperature, η is the viscosity of the solvent, and RH is the hydrodynamic radius.

### 2.11. Antimicrobial Activity

The antimicrobial activities of the Poly(alkylene biguanide) were tested against *P. aeruginosa*, *S. aureus* and *C. albicans* using the broth microdilution method adapted from CLSI guidelines (M07 for bacteria and M27 for yeasts) [28,29].

*S. aureus* and *P. aeruginosa* were cultured on tryptic soy agar (TSA), while *C. albicans* was cultured on Sabauroud dextrose agar (SDA). All cultures were incubated at 35 °C for 18–24 h. Overnight cultures were adjusted in sterile phosphate-buffered saline to an OD600 of 0.09–0.1 and then diluted 1:100 in double-strength tryptic soy broth (TSB) for bacteria or Sabouraud dextrose broth (SDB) for *C. albicans*, resulting in an approximate final inoculum of ~5 × 10^5^ CFU/mL for bacteria or ~10^3^ CFU/mL for C. albicans in the assay wells.

The polymers were serially diluted in sterile distilled water in 96-well microplates. Two concentration ranges were prepared: 250–0.24 µg/mL and 100–0.1 µg/mL. Each well contained 100 µL of polymer solution followed by 100 µL of microbial inoculum (final volume: 200 µL). Wells containing inoculum without polymer served as positive controls, while sterile TSB or SDB served as negative controls. Bacterial plates were incubated at 35 °C for 24 h, and *C. albicans* plates were incubated for 48 h. PHMB was used as a reference compound.

The minimum inhibitory concentration (MIC) was defined as the lowest concentration at which no visible turbidity was observed. Minimum bactericidal concentration (MBC) and minimum fungicidal concentration (MFC) values were determined by subculturing 10 µL from wells without visible growth onto TSA or SDA plates. Plates were incubated at 35 °C for 24 h, and the absence of colony formation indicated bactericidal or fungicidal activity.

## 3. Results

### 3.1. Physical Properties

**Figure 3:**
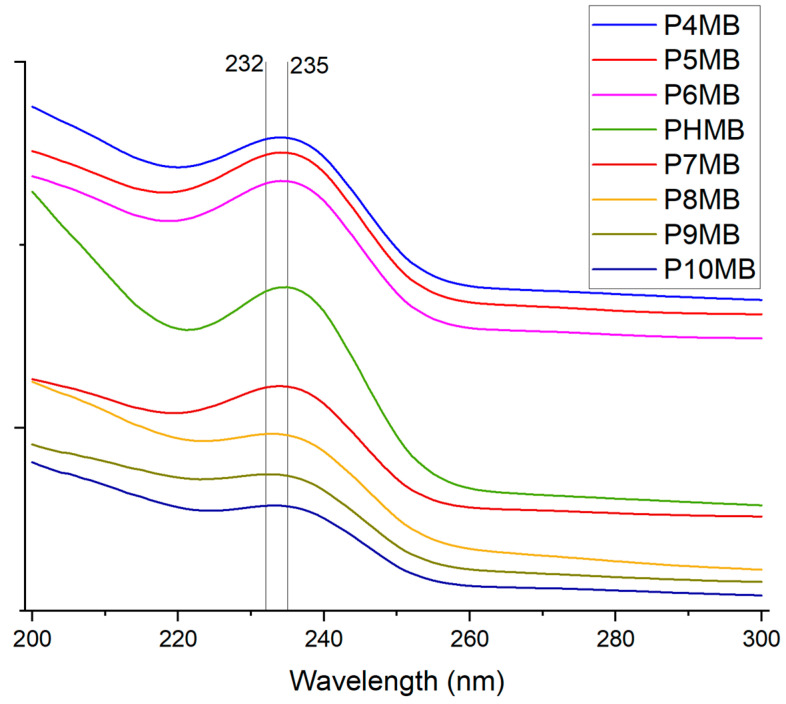
Stacked UV spectra of Poly(alkylene biguanide) (P4MB–P10MB) and marketed PHMB in aqueous solution at room temperature, recorded over the wavelength range 200–300 nm. Spectra are presented in a stacked format for visual comparison; absorbance scales differ.

The poly(alkyl biguanide) polymers exhibited a characteristic absorption peak at λ = 232 to 235 nm (Table 1, Figure 3). Solubility of the poly(alkyl biguanide) polymers decreased with increasing alkyl chain length, ranging from 17.16% for P4MB to 0.19% for P10MB. Marketed PHMB formed a highly viscous solution, with solubility exceeding 40%. The predicted refractive index decreased as the alkyl chain length of the Poly(alkylene biguanide) increased. In contrast, the calculated LogP values increased from −2.05 for P4MB to +1.12 for P10MB, as summarised in Table 1.

### 3.2. FTIR

**Figure 4:**
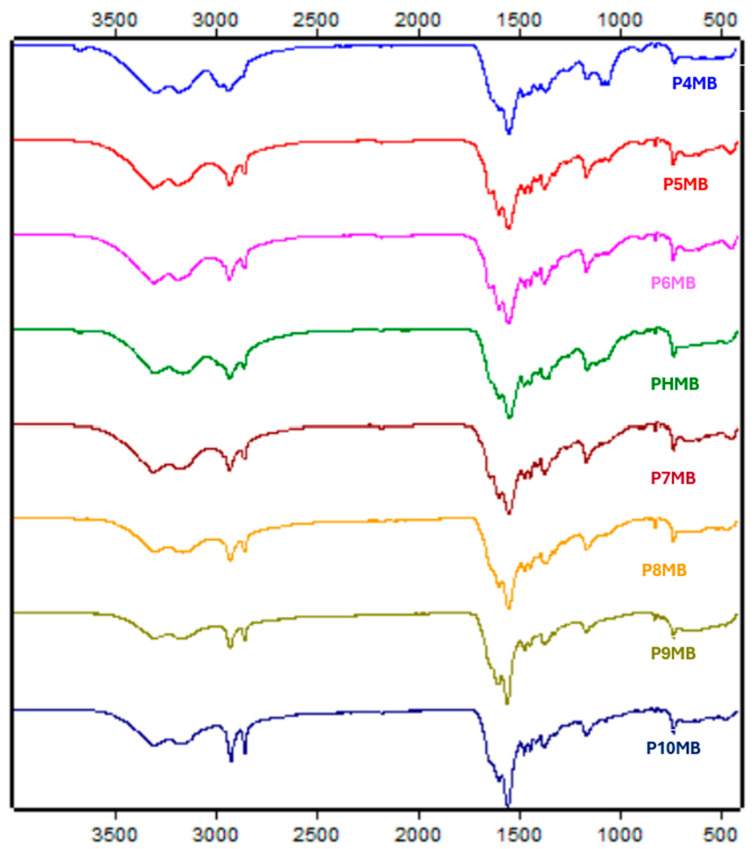
FT-IR spectra of solid Poly(alkylene biguanide) with alkyl chain lengths from C4 to C10 (P4MB, P5MB, P6MB, marketed PHMB, P7MB, P8MB, P9MB and P10MB), recorded in the range 400–4000 cm^−1^.

FTIR spectra of the Poly(alkylene biguanide) showed similar characteristic peaks (Table S1, Figure 4) NH, =NH stretching at ~3300 and 3170 cm^−1^, -CH_2_ stretching at ~2920, 2855 cm^−1^ and overlap ped peaks of >C=N stretching and NH_2_ bending at ~1624, 1587 and 1535 cm^−1^.

### 3.3. DLS

**Figure 5:**
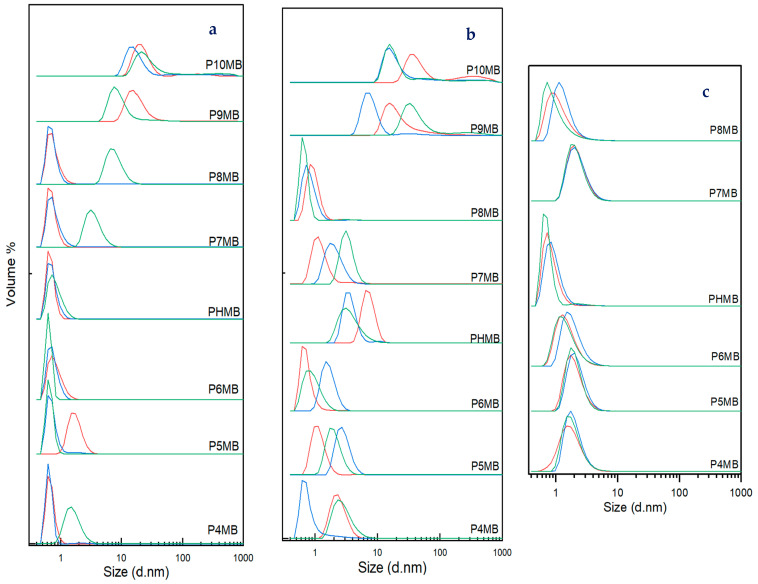
Particle size distribution by volume in water for solid Poly(alkylene biguanide) (P4MB, P5MB, P6MB, marketed PHMB, P7MB, P8MB, P9MB, P10MB) at 25 °C, measured at concentrations of (**a**) 0.1 mg/mL, (**b**) 1 mg/mL and (**c**) 10 mg/mL using dynamic light scattering (Run 1 (red), Run 2 (blue), and Run 3 (green)).

Based on the diffraction light scattering (DLS), the volume and size distribution of polymers P4MB to P8MB were measured at concentrations of 0.1, 1 and 10 mg/mL. The results are presented in Figure 5 and Figure S1 and Table S2. At 0.1 mg/mL, the average diameters of polymers up to P8MB were ≤5 nm, with some variation observed within triplicates. Increasing the concentration to 1 mg/mL led to a slight increase in average diameter, except for P8MB; however, all values remained ≤5 nm. At a concentration of 10 mg/mL, the average diameters were equal to or smaller than those observed at 1 mg/mL, with a maximum diameter of ≤2.3 nm. P9MB and P10MB were not measured at 10 mg/mL due to solubility limitations. Unlike the more hydrophilic polymers, the hydrophobic polymers exhibited larger average diameters of 20 nm at 0.1 mg/mL and 38 nm at 1 mg/mL for P9MB, and 68 nm at 0.1 mg/mL and 84 nm at 1 mg/mL for P10MB.

### 3.4. NMR and DOSY

**Figure 6:**
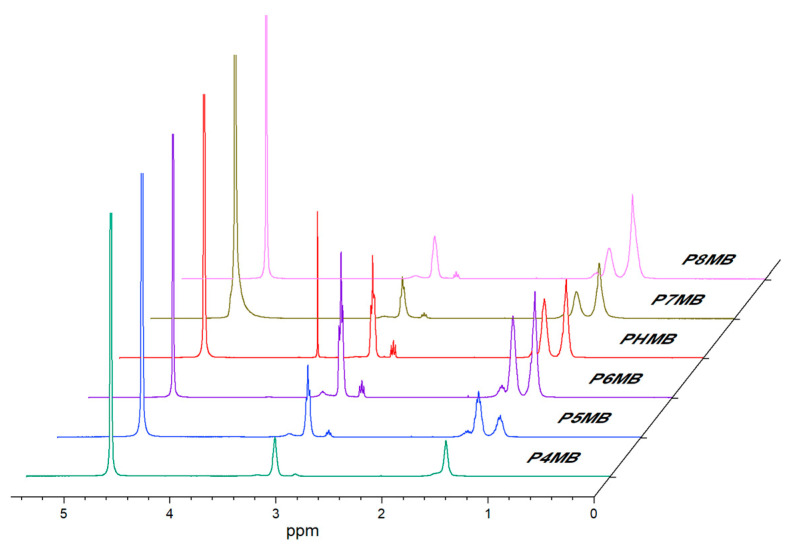
H NMR spectra of Poly(alkylene biguanide) with alkyl chain lengths from C4 to C8 (P4MB, P5MB, P6MB, marketed PHMB, P7MB, and P8MB), recorded in D_2_O at 25 °C.

The ^1^H NMR (D_2_O) spectra of the polymers (Figure 6, Table S3) show the residual solvent peak at δ 4.7 (HDO). P4MB gave peaks at δ 1.5 (4H, –CH_2_–) and δ 3.15 (4H, –CH_2_–N–). Polymers with C_5_–C_8_ alkyl chains exhibited peaks at approximately δ 1.5–1.6 (–CH_2_, 6–12H) and δ 3.1 (4H, –CH_2_–N–). Due to limited solubility in water, P9MB and P10MB were analysed in DMSO-d_6_ (residual solvent peak at δ 2.50), along with other polymers, to enable direct comparison (Figure S2). P4MB showed signals at δ 1.49 (4H, –CH_2_–) and δ 3.11 (4H, –CH_2_–N–). Polymers with C_5_–C_10_ alkyl chains displayed peaks at δ 1.27 and 1.44 (–CH_2_–, 6–16H). A signal at ~δ 3.3 was attributed to residual water in DMSO-d_6_. Broad singlets observed at δ 6.5–8.6 corresponded to NH groups; these signals were visible in DMSO-d_6_ but exchanged in D_2_O [30]. Marketed PHMB exhibited an additional singlet at δ 3.5, consistent with the presence of O-methylene groups.

The molecular weights of the polymers were calculated using end-group analysis, where the end-groups were amine, guanidine or nitrile [31]. The molecular weights of the synthesised polymers were between 2 kDa and 3 kDa in D_2_O. The calculated molecular weight in DMSO-d_6_ was higher compared with that in D_2_O, with values ranging from 2.5 KDa to 4 KDa (Table 1, Figures S3 and S4).

The diffusion coefficients obtained using DOSY NMR were used to estimate the distribution of hydrodynamic diameters and the apparent polydispersity (PDI) of the polymers (Table 2). The polydispersity values were calculated from the diffusion coefficients of the end-groups (extremities) and the polymer backbone using the barycentric approach. A fractal dimension of 7/3 (~2.3) was applied, representing the midpoint between 5/3 for solvated chains and 3 for compact structures. This choice reflects the observation that the polymers are soluble in water and form single-chain folded conformations [26].

PDI values indicated that the solvent plays a crucial role in the dispersibility of the polymers, as solubility varies between solvents. This suggests that solvent polymer interactions influence the degree of chain aggregation or unfolding during measurement. P4MB and P5MB showed consistent PDI values in both solvents, likely due to their shorter hydrophobic chains. In contrast, no clear trends were observed for polymers with longer alkyl chains, possibly reflecting greater sensitivity to solvent polarity and hydrophobic interactions. These variations suggest that the solvent environment may alter the conformation of the polymers.

The diffusion rates in D_2_O were faster than those in DMSO-d6. Accordingly, the hydrodynamic volume was smaller in D_2_O, with an approximate diameter of 4 nm, compared with 5–6 nm in DMSO-d_6_. Furthermore, in D_2_O, most polymers exhibited one main fraction and a secondary, smaller fraction corresponding to larger diameters. Marketed PHMB showed a smaller hydrodynamic diameter.

### 3.5. Antimicrobial Activity

**Figure 7:**
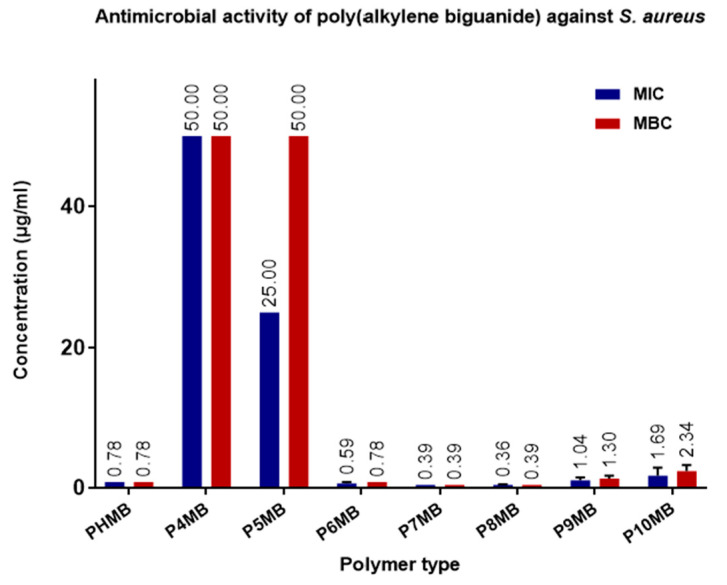
Minimum inhibitory concentration (MIC) and minimum bactericidal concentration (MBC) of Poly(alkylene biguanide) against *S. aureus* P4MB to P10MB (*n* = 6, mean ± SD). MIC and MBC values (µg/mL) are displayed at the top of the bars.

Figure 7 shows the activity of Poly(alkylene biguanide) against *S. aureus*. Although P7MB and P8MB exhibited higher activity, the difference was not statistically significant compared with the marketed PHMB. The least active compounds are P4MB and P5MB.

**Figure 8:**
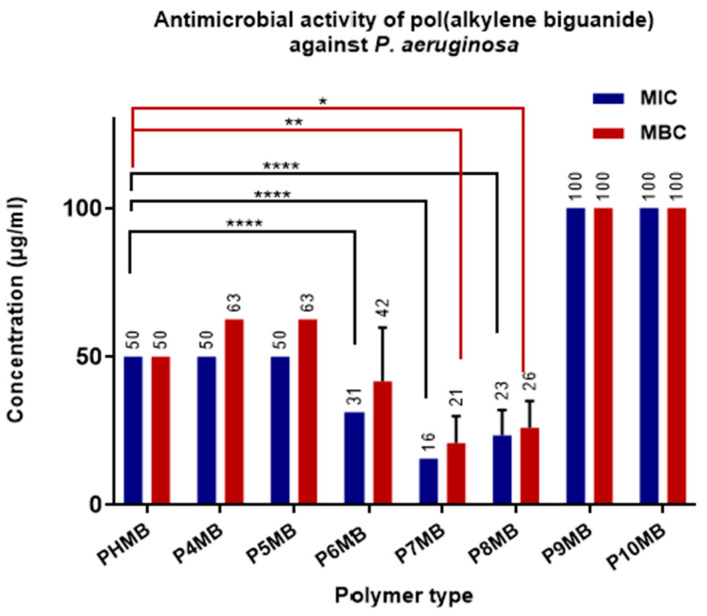
Minimum inhibitory concentration (MIC) and minimum bactericidal concentration (MBC) of Poly(alkylene biguanide) P4MB to P10MB against *P. aeruginosa* (*n* = 6, mean ± SD). MIC and MBC values (µg/mL) are displayed at the top of the bars. Significance levels are indicated as follows: * (*p* < 0.05), ** (*p* < 0.01), and **** (*p* < 0.0001).

As presented in Figure 8, P7MB and P8MB showed the highest antimicrobial activity against *P. aeruginosa*, with MIC values of 15.0 and 23.4 µg/mL, respectively, compared with 50 µg/mL for PHMB. These differences were highly significant (*p* < 0.0001). Interestingly, P6MB also showed a similar level of significance, with an MIC value of 31.25 µg/mL. The MBC values of P7MB (20.8 µg/mL) and P8MB (26.0 µg/mL) were also statistically significant, with *p*-values of <0.01 and <0.05, respectively, compared with the marketed PHMB MBC of 50 µg/mL. However, the MBC of P6MB was not statistically significant compared with that of PHMB. P9MB and P10MB were the least active polymers.

**Figure 9:**
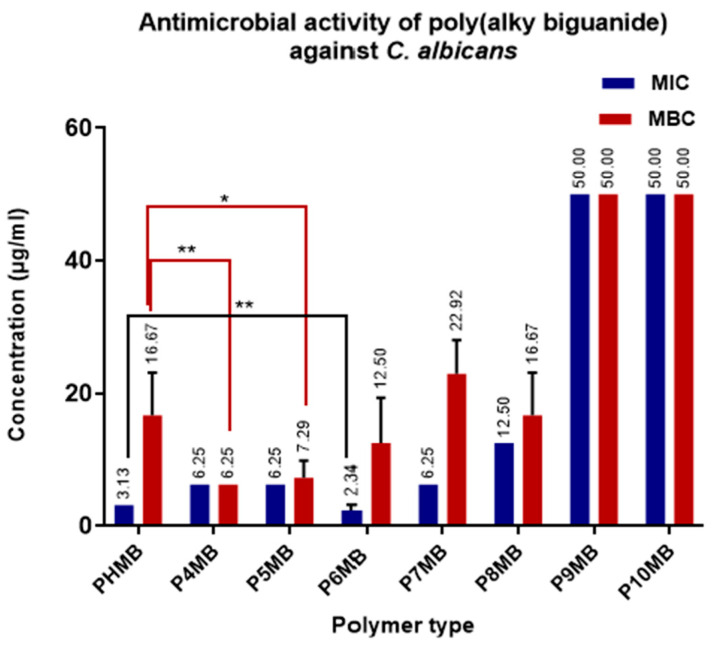
Minimum inhibitory concentration (MIC) and minimum fungicidal concentration (MFC) of Poly(alkylene biguanide) P4MB to P10MB against *C. albicans* (*n* = 6, mean ± SD). MIC and MBC values (µg/mL) are displayed at the top of the bars. Significance levels are indicated as follows: * (*p* < 0.05), and ** (*p* < 0.01).

The activity of Poly(alkylene biguanide) against *C. albicans* is shown in Figure 9. P6MB inhibited the growth of *C. albicans* at a concentration of 2.34 µg/mL, compared with PHMB at 3.13 µg/mL, with a statistically significant difference (*p* < 0.001). Although the inhibitory concentrations of P4MB and P5MB were 6.25 µg/mL, which is higher than that of marketed PHMB, both showed greater fungicidal potency, with MFCs of 6.25 µg/mL for P4MB and 7.29 µg/mL for P5MB, compared with 16.67 µg/mL for PHMB. These differences were statistically significant, with levels of *p* < 0.05 and < 0.001, respectively. The least active compounds were the lipophilic P9MB and P10MB, with MIC and MFC of 50 µg/mL.

## 4. Discussion

The solubility of the Poly(alkylene biguanide) is consistent with their chemical structures, which decreases with increasing alkyl chain length, owing to the enhanced hydrophobicity associated with longer alkyl chains. This trend correlates with the higher calculated Log*P* values. The solubility of P6MB in distilled water is approximately 11%, whereas the solubility of marketed PHMB could not be quantitatively measured because the polymer forms a highly viscous, three-dimensional network that prevents analysis. Nevertheless, it is documented in the literature that the solubility of PHMB in distilled water exceeds 40% [31]. Both P6MB and PHMB are expected to have identical chemical structures; however, the ^1^H NMR spectrum of PHMB shows an additional peak at 3.5 ppm, potentially attributed to an O–CH_2_– moiety, which could have influenced the solubility [10].

The polycationic nature of the polymers, which remain highly ionised at physiological pH and do not exist in a neutral state, makes the experimental determination of log*P* impractical; therefore, computational estimation remains the only feasible approach. The European Commission’s Scientific Committee on Consumer Safety has reported a log*D* value of −2.3 for PHMB at pH 7.4, owing to the strongly ionisable nature of PHMB [7,32].

The calculated refractive index for both PHMB and P6MB is 1.595, which is in close agreement with the experimental value of 1.5486 [31]. The calculated values were employed in the DLS analysis to ensure consistency across comparisons. The observed decrease in refractive index with increasing alkyl chain length may be attributed to enhanced chain flexibility and reduced molecular packing [33,34]. The UV spectra show a maximum absorption at 232–234 nm for all polymers, attributed to the π → π transition of the −C=N− group in the biguanide moiety [35]. The maximum UV absorption for polymers with 4 to 7 carbon atoms is approximately 234 nm. A slight hypsochromic (blue) shift is generally observed with increasing alkyl chain length from 7 to 9 carbon atoms, likely due to increased spatial separation between chromophores, which reduces electronic coupling. In contrast, both P9MB and P10MB exhibit a hypochromic effect, evidenced by a decrease in absorption intensity. Additionally, a bathochromic (red) shift is observed from P9MB to P10MB, suggesting enhanced electronic coupling between chromophores because of increased chain flexibility [36].

The FTIR spectra of the polymers displayed similar characteristic peaks with N−H, =N−H stretching at ~3300 and 3170 cm^−1^, −CH_2_ stretching at ~2920, 2855 cm^−1^ and overlapped peaks of >C=N stretching and NH_2_ bending at ~1624, 1587 and 1535 cm^−1^ (Figure 4 and Table S1). These results are consistent with previous findings related to PHMB [10]. The length of the alkyl chain did not significantly affect the FTIR spectra [37]. Successful polymerisation was evidenced by the disappearance of the C≡N peak at ~2176 cm^−1^ [38]. The ^1^H NMR spectra of the synthesised polymers recorded in D_2_O and DMSO-d_6_ showed almost identical chemical shifts, suggesting a limited solvent influence on proton environments.

DLS was used to investigate the behaviour of the polymers in water and to determine their hydrodynamic diameter. While both DLS and DOSY NMR can be used to measure hydrodynamic size, DLS is more sensitive to the presence of stable aggregates, whereas DOSY NMR is sensitive to molecular diffusion and reflects the relative proportions of different fractions in solution [39]. At low concentrations (0.1 and 1 mg mL^−1^), DLS measurements revealed slight fluctuations in the apparent hydrodynamic diameter of the Poly(alkylene biguanide), even when the same sample was analysed in triplicate (Figure 5, Table S2). This behaviour is attributed to the dynamic folding and unfolding of the polymer chains in aqueous solution. However, at higher concentrations, all polymers exhibited a more consistent diameter. The behaviour of the polymers may be explained by the folding of individual chains into hairpin-like structures, suggesting that the self-association process is reversible. This observation has been reported previously for PHMB [40]. Such conformational dynamics likely arise from the balance between electrostatic repulsion and hydrophobic interactions along the polymer backbone, which stabilises at higher concentrations. P9MB and P10MB could not be assessed at 10 mg/mL due to their poor water solubility. At lower concentrations, P9MB and P10MB exhibited larger hydrodynamic volumes, with average diameters of approximately 20 nm and 67 nm at 0.1%, and 38 nm and 83 nm at 1%, respectively. This increase is attributed to the self-aggregation of the polymers resulting from the increased hydrophobic chain lengths [41]. However, their sizes in DOSY NMR in DMSO-d_6_ are within the range of other less hydrophobic synthesised polymers of around 4 nm.

The hydrodynamic diameter of the polymers was calculated from the diffusion coefficients using the Stokes–Einstein equation. Although solvent viscosity was taken into account, the apparent diffusion coefficients were lower in DMSO-d_6_ than in D_2_O, which is attributed to stronger solvent–polymer interactions in DMSO-d_6_ [27]. This can be explained by the formation of strong hydrogen bonds between D_2_O and the biguanide groups, together with hydrophobic interactions among the alkyl segments of the polymer, which promote intramolecular folding and lead to a more compact structure. In contrast, in DMSO-d_6_, dipole–dipole interactions predominate; these interactions involve both the biguanide and alkyl groups, weakening intra-chain associations resulting in a more extended conformation and a larger hydrodynamic diameter [42]. P4MB, P6MB, and P7MB show less than 1% fractions of bigger size diameters that disappear in DMSO-d_6_. This may be linked to the formation of stable self-association in D_2_O. However, marketed PHMB exhibited two distinct volume fractions in both DMSO-d_6_ and D_2_O, as did P9MB in DMSO-d_6_ (Table 2).

The activity of antimicrobial polymers is influenced by their charge density, hydrophobic groups, and molecular weight [41]. Poly(alkyl biguanide) polymers exhibited varying antimicrobial activity against the tested microorganisms, indicating that their structural differences influence their biological activities. P4MB and P5MB, which exhibit relatively high cationic charge density, showed superior biocidal activity against *C. albicans* compared with the other poly(alkyl biguanide) polymers. In a separate study, the minimum inhibitory concentration (MIC) of PHMB against *C. albicans* was reported to be 1.25–2.5 µg mL^−1^, which is comparable to our findings (Figure 9). The proposed mechanism of action involves destabilisation of the cell membrane through potassium efflux and the formation of pores approximately 2.3–3.3 nm in diameter [43]. Interestingly, this pore size is comparable to the hydrodynamic diameter of the polymers determined in our study, which may indicate that the polymers can penetrate the cell membrane. However, it remains unclear why P4MB and P5MB exhibited higher fungicidal activity. This finding is unexpected, as fungal cells possess fewer anionic surface sites than bacterial cells.

P4MB and P5MB showed the lowest activity against *S. aureus*, whereas P7MB and P8MB exhibited greater activity against both *P. aeruginosa* and *S. aureus*, being particularly potent against *S. aureus*. The anionic surface charge of *S. aureus* is predominantly attributed to bacterial wall teichoic acid and lipoteichoic acid. Several cationic polymers, including polyethylenimine and guanidine-based copolymers, have been shown to interact with these acids, leading to membrane destabilisation and subsequent permeabilisation [44,45]. Given the structural similarity of marketed PHMB to other biguanide and polycationic systems, it is plausible that PHMB and related Poly(alkylene biguanide) polymers engage in similar interactions with teichoic acids. It has also been reported that PHMB can enter *S. aureus* cells and selectively condense bacterial chromosomes [46]. Moreover, the activity of PHMB against methicillin-resistant *S. aureus* (MRSA) is equivalent to its activity against methicillin-sensitive *S. aureus* (MSSA) [47].

The reported MIC values of PHMB against different clinical isolates of *P. aeruginosa* strains range from 15.6 to 31.25 µg mL^−1^, which is comparable to our findings (Figure 8). This activity is likely attributed to interactions with the lipopolysaccharide layer, leading to disruption of the cell membrane [48]. The immobilisation of the poly(alkyl biguanide) polymers on the microbial surface and their subsequent penetration into the cell membrane are enhanced by the hydrophobic alkyl chains.

P9MB and P10MB exhibited lower activity against both *P. aeruginosa* and *C. albicans* than the other polymers, most likely due to aggregate formation resulting from their higher hydrophobicity, as evidenced by DLS. Such aggregation can reduce the effective interaction between the polymer chains and microbial cell membranes [41]. However, the higher charge density associated with the biguanide moiety in both P4MB and P5MB resulted in minimal antibacterial activity against *S. aureus*.

## 5. Conclusions

The length of the alkyl chain in polymeric biguanides has a significant impact on antimicrobial activity. Chains with 7 or 8 carbon atoms exhibited the highest antibacterial activity, whereas chains with 4 or 5 carbon atoms showed the greatest fungicidal activity. Variations in hydrophobicity and hydrophilicity associated with different chain lengths open the door to further investigate the mechanisms underlying the antimicrobial activity of PHMB and other polymeric biguanides. Such studies may lead to the identification of new drug targets for antimicrobials and potentially help reduce the risk of antimicrobial resistance, as PHMB has not been reported to induce resistance. In our future work, we aim to further explore the mechanism of antimicrobial activity alongside cytotoxicity to design drug delivery systems suitable for human use.

## Figures and Tables

**Table 1:** Physical properties of the Poly(alkylene biguanide).

Poly(alkylene biguanide)	Solubility (Water)	Clog*P* (ChemDraw)	Predicted Refractive Index	λ_max_ nm
P4MB	17.16 ± 1.07%	−2.05	1.678	234.0
P5MB	9.61 ± 0.47%	−1.52	1.630	234.2
P6MB	11.35 ± 1.14%	−0.99	1.595	234.3
P7MB	6.33 ± 0.58%	−0.47	1.572	234.6
P8MB	4.25 ± 0.20%	+0.64	1.555	234.0
P9MB	0.53 ± 0.02%	+0.59	1.544	232.5
P10MB	0.19 ± 0.01%	+1.12	1.536	232.4

**Table 2:** Polydispersity index (PDI), fractional hydrodynamic diameter, degree of polymerisation (*n*), and calculated molecular weight for Poly(alkylene biguanide).

Polymer/Solvent	PDI	Fraction A		Fraction B		Degree of Polymerisation (*n*)	Calculated Molecular Weight
		Diameter (nm)	Percent %	Diameter (nm)	Percent %		
P4MB/D_2_O	1.28	4.11	99.6%	33.79	0.4%	17	2895
P5MB/D_2_O	1.65	4.81	100.0%			14	2562
P6MB/D_2_O	1.43	4.54	99.1%	18.22	0.9%	10	2205
PHMB/D_2_O	1.64	2.57	73.8%	7.61	26.2%	8	1784
P7MB/D_2_O	1.45	3.41	99.8.0%	18.49	0.2%	11	2394
P8MB/D_2_O	1.06	3.34	100.0%			12	2985
P4MB/DMSO	1.32	5.44	100.0%			22	3639
P5MB/DMSO	1.69	6.15	100.0%			15	2753
P6MB/DMSO	1.09	5.30	100.0%			12	2434
PHMB/DMSO	1.70	3.67	81.5%	10.21	18.5%	9	1907
P7MB/DMSO	1.12	4.83	100.0%			13	2827
P8MB/DMSO	1.58	5.14	100.0%			14	3307
P9MB/DMSO	1.95	5.25	93.5%	19.50	6.5%	17	4174
P10MB/DMSO	1.68	5.96	100.0%			15	4024

## Data Availability

Data are submitted as Supplementary Materials.

## Supplementary Materials

The following supporting information can be downloaded at https://www.mdpi.com/article/10.3390/polym18030390/s1. Figure S1: Particle size distribution (Dynamic Light Scattering, DLS) by volume in water for solid poly(alkyl biguanides) P4MB (a), P5MB (b), P6MB (c), marketed poly(hexamethylene biguanide) (PHMB) (d), P7MB (e), P8MB (f), P9MB (g) and P10MB (h) at 25 °C, measured at concentrations of (1) 0.1 mg/mL, (2) 1 mg/mL, (3) 10 mg/ml. Figure S2: 1H NMR spectra of poly(alkyl biguanides) with alkyl chain lengths from C4 to C10 (P4MB, P5MB, P6MB, P7MB, P8MB, P9MB, and P10MB), including marketed poly(hexamethylene biguanide) (PHMB), recorded in DMSO-d_6_ at 25 °C. Figure S3: ^1^H NMR spectra of poly(alkylene biguanide) samples (P4MB–P8mb) in D_2_O (400 MHz, 25 °C). Integration values were used for end-group analysis to calculate molecular weight. (a) P4MB, (b) P5MB, (c) P6MB, (d) PHMB, (e) P7MB, (g) P8MB. Figure S4: ^1^H NMR spectra of poly(alkylene biguanide) samples (P4MB–P8MB) in DMSO-d_6_ (400 MHz, 25 °C). Integration values were used for end-group analysis to calculate molecular weight. (a) P4MB, (b) P5MB, (c) P6MB, (d) PHMB, (e) P7MB, (f) P8MB, (g) P9MB, (h) P10MB. Table S1: FTIR characteristic peaks of poly(alkylene biguanide) polymers. Table S2: Volume diameters of the poly(alkylene biguanide) polymers at 0.1, 1, and 10 mg/mL at 25 °C, measured using Dynamic Light Scattering (DLS). Table S3: 1H NMR chemical shifts for poly(alkylene biguanide) polymers in D_2_O and DMSO-d_6_. Table S4: Fractional diffusion coefficient of poly(alkylene biguanide) polymers obtained from 2D DOSY. Table S5: 2D DOSY spectra and Inverse Laplace Transform figures for poly(alkylene biguanide) polymers in D_2_O. Table S6: 2D DOSY spectra and Inverse Laplace Transform figures for poly(alkylene biguanide) polymers in DMSO-d_6._
