# Supplementary material for: Alkyl Chain Length Governs Structure, Conformation and Antimicrobial Activity in Poly(alkylene biguanide)
Source: Polymers (Basel). 2026 Feb 1;18(3):390. doi: 10.3390/polym18030390 (PMC12899342; doi:10.3390/polym18030390)
Supplement: Supplementary file 1 [file polymers-18-00390-s001.zip › polymers-4128989-supplementary.pdf]

Alkyl Chain Length Governs Structure, Conformation and Antimicrobial Activity in Poly(Alkylene Biguanide)

Enas Al-Ani [1,*], Khalid Doudin [2], Andrew J. McBain [1], Zeeshan Ahmad [1] and Sally Freeman [1]

[1] Division of Pharmacy and Optometry, University of Manchester, Manchester M13 9PL, UK
[2] Department of Mathematical and Physical Sciences, The University of Sheffield, Sheffield S10 2TN, UK
* Correspondence: enas.al-ani@manchester.ac.uk

Table S1 *FTIR characteristic peaks of poly(alkylene biguanide) polymers.*

Polymer	-NH, =NH str	-CH_2 stretching	>C=N stretching, NH_2 bending		
**P4MB**	3294, 3176	2971, 2924	1622	1588	1540
**P5MB**	3304, 3185	2926, 2851	1635	1589	1541
**P6MB**	3304, 3185	2926, 2851	1635	1589	1542
**PHMB**	3296, 3161	2927, 2856	1626	1589	1538
**P7MB**	3303, 3184	2926, 2851	1634	1589	1540
**P8MB**	3297,3160	2921, 2849	1622	1589	1539
**P9MB**	3299, 3177	2922, 2849	1638	1594	1549
**P10MB**	3305, 3176	2919, 2848	1634	1589	1548

Table S2 *Volume diameters of the poly(alkylene biguanide) polymers at 0.1, 1, and 10 mg/mL at 25 °C, measured using Dynamic Light Scattering (DLS).*

Polymer	Concentration	Volume diameter (nm)			Average	SD
		Replicate 1	Replicate 2	Replicate 3		
P4MB	0.1 mg/ml	0.69	0.69	1.62	1.00	0.54
P5MB		1.82	0.76	0.67	1.08	0.64
P6MB		0.74	0.63	0.90	0.76	0.13
PHMB		0.68	0.73	0.82	0.74	0.07
P7MB		0.72	0.77	4.49	1.99	2.17
P8MB		0.76	0.71	13.34	4.94	7.28
P9MB		13.88	31.06	16.18	20.37	9.33
P10MB		62.23	54.73	86.67	67.88	16.70
P4MB	1 mg/ml	2.49	0.86	3.17	2.17	1.19
P5MB		1.20	2.95	2.08	2.08	0.87
P6MB		0.75	1.70	0.94	1.13	0.50
PHMB		8.07	4.23	4.77	5.69	2.08
P7MB		1.29	2.12	3.45	2.28	1.09
P8MB		0.99	0.84	0.73	0.85	0.13
P9MB		32.31	11.63	71.08	38.34	30.18
P10MB		127.40	60.41	62.75	83.52	38.02
P4MB	10 mg/ml	1.81	2.02	1.92	1.92	0.11
P5MB		2.05	2.22	2.06	2.11	0.10
P6MB		1.54	1.89	1.52	1.65	0.21
PHMB		0.86	0.96	0.80	0.87	0.08
P7MB		2.27	2.31	2.19	2.26	0.06
P8MB		1.19	1.40	1.04	1.21	0.18

***Figure S1** Particle size distribution (Dynamic Light Scattering, DLS) by volume in water for solid poly(alkyl biguanides) P4MB (a), P5MB (b), P6MB (c), marketed poly(hexamethylene biguanide) (PHMB) (d), P7MB (e), P8MB (f), P9MB (g) and P10MB (h) at 25 °C, measured at concentrations of (1) 0.1 mg/mL, (2) 1 mg/mL, (3) 10 mg/ml*

(a1) P4MB 0.1 mg/ml

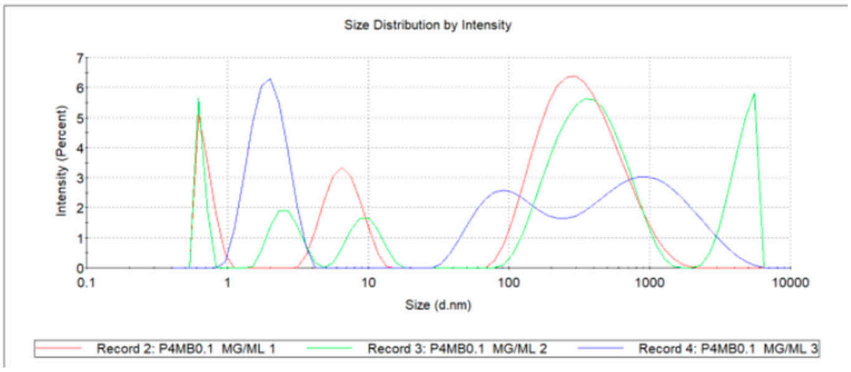

(a2) P4MB 1 mg/ml

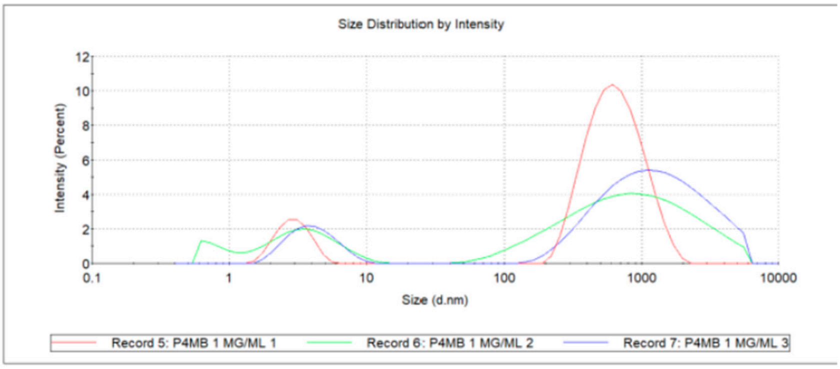

(a3) P4MB 10 mg/ml

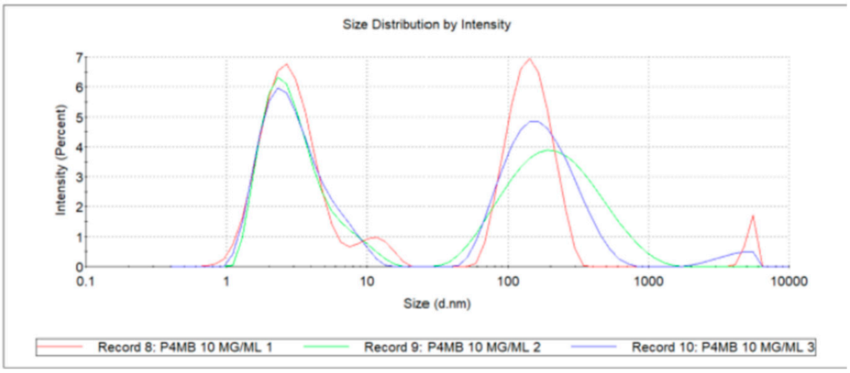

(b1) P5MB 0.1 mg/ml

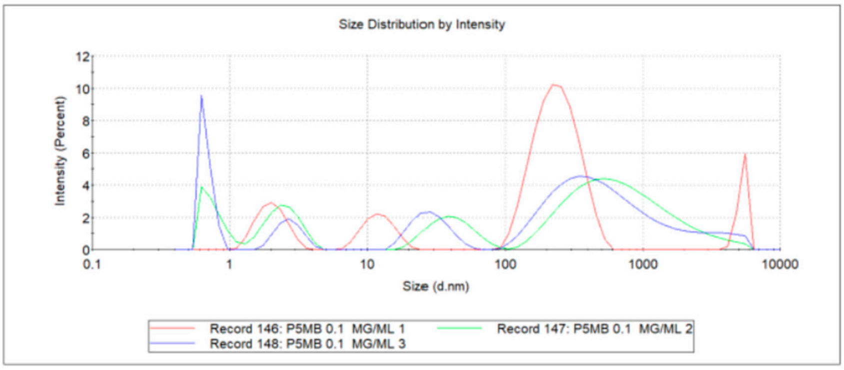

(b2) P5MB 1 mg/ml

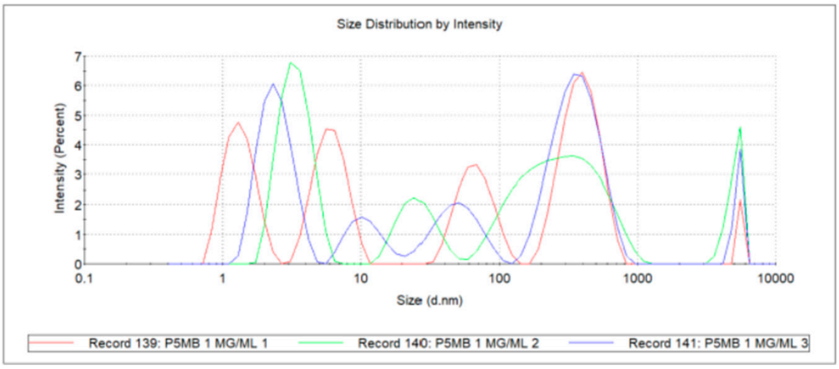

(b3) P5MB 10 mg/ml

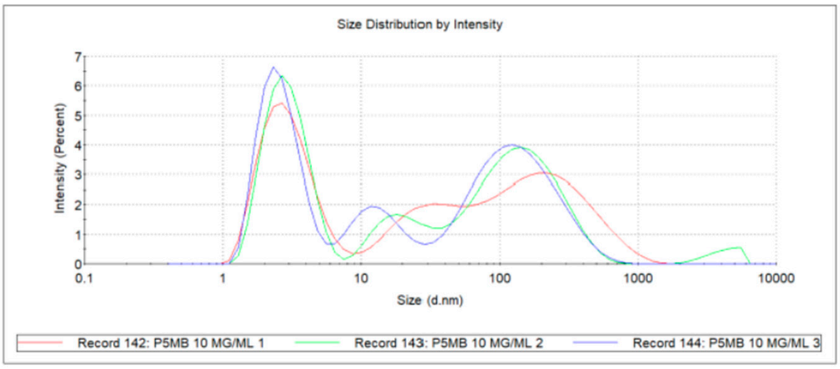

(c1) P6MB 0.1 mg/ml

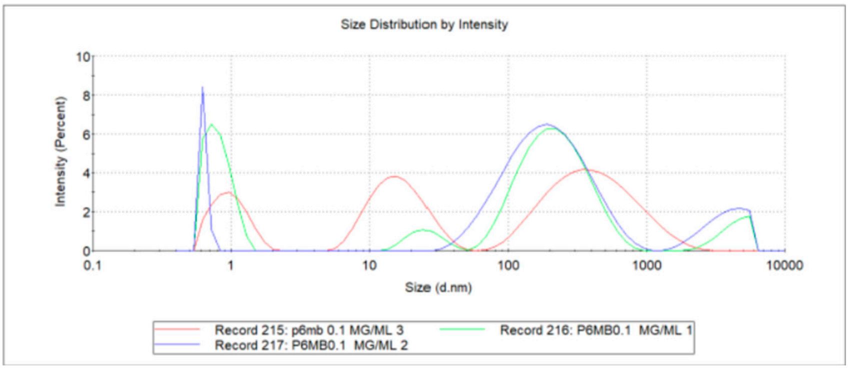

(c2) P6MB 1mg/ml

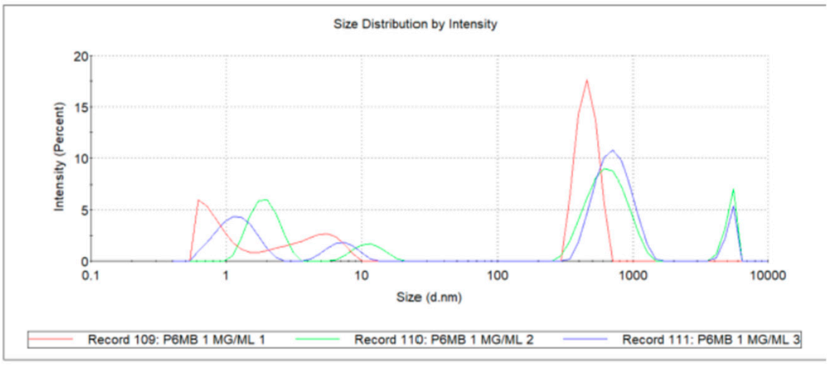

(c3) P6MB 10 mg/ml

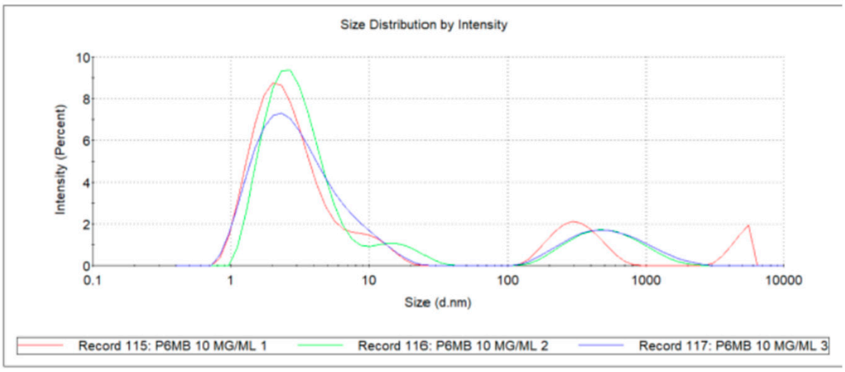

(d1) PHMB 0.1 mg/ml

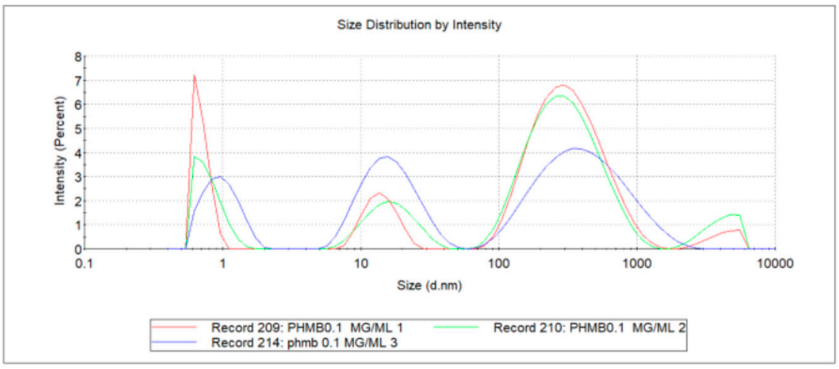

(d2) PHMB 1mg/ml

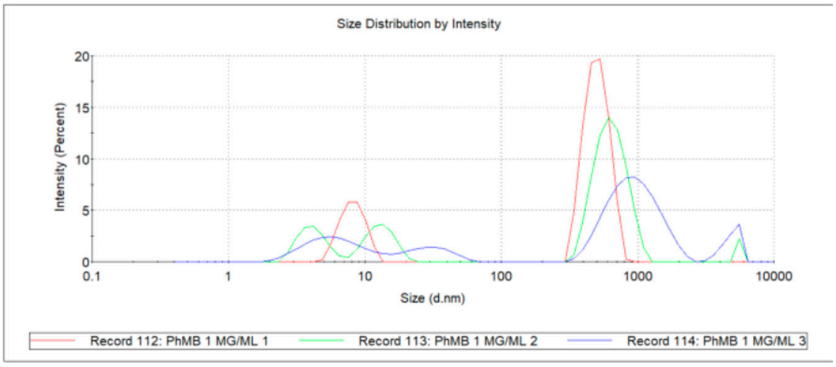

(d3) PHMB 10 mg/ml

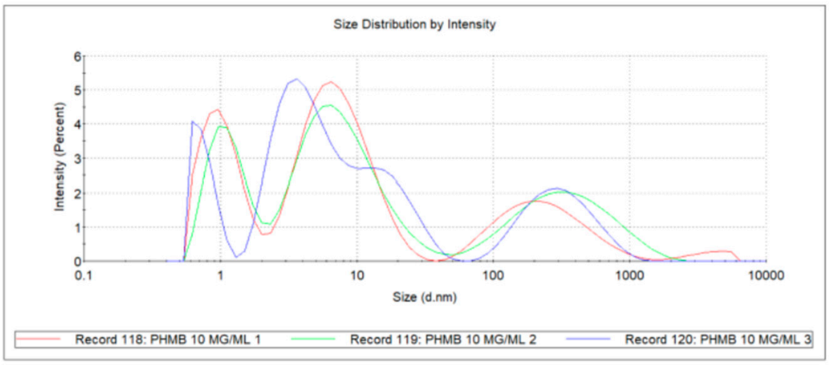

(e1) P7MB 0.1 mg/ml

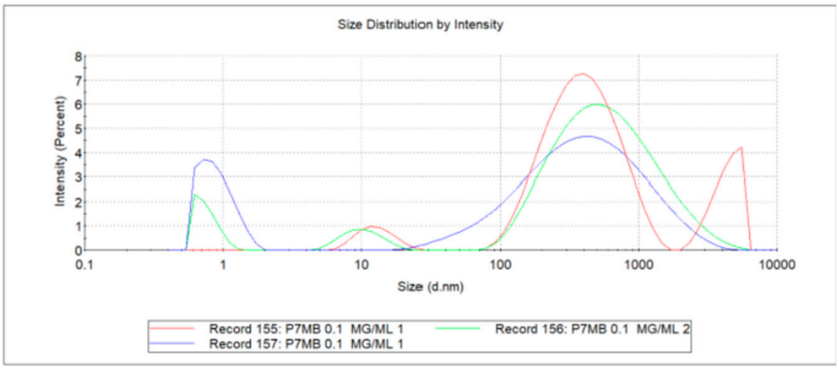

(e2) P7MB 1 mg/ml

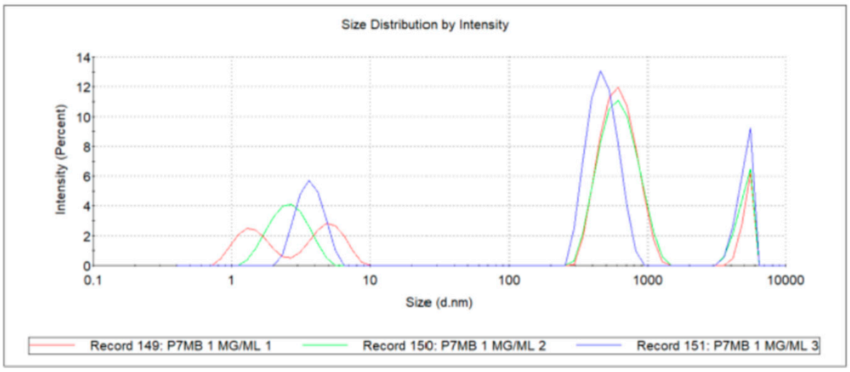

(e3) P7MB 10 mg/ml

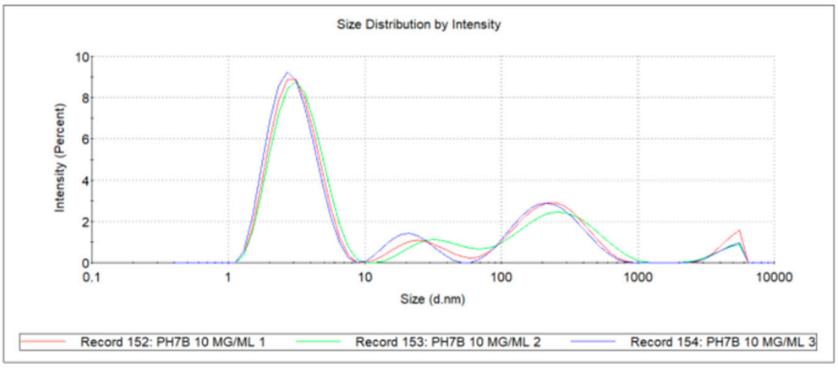

(f1) P8MB 0.1 mg/ml

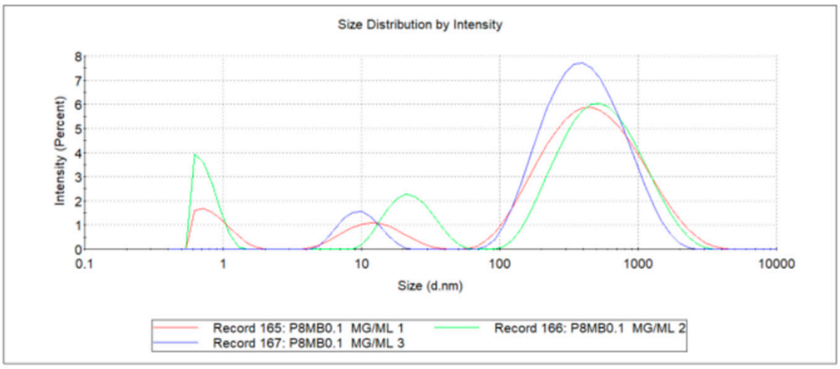

(f2) P8MB 1 mg/ml

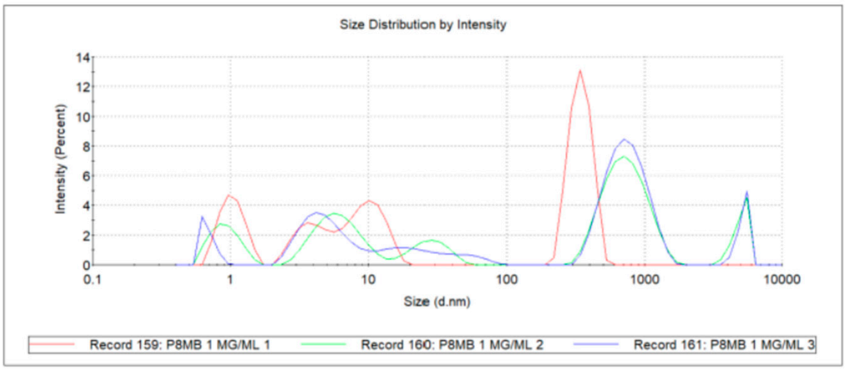

(f3) P8MB 10 mg/ml

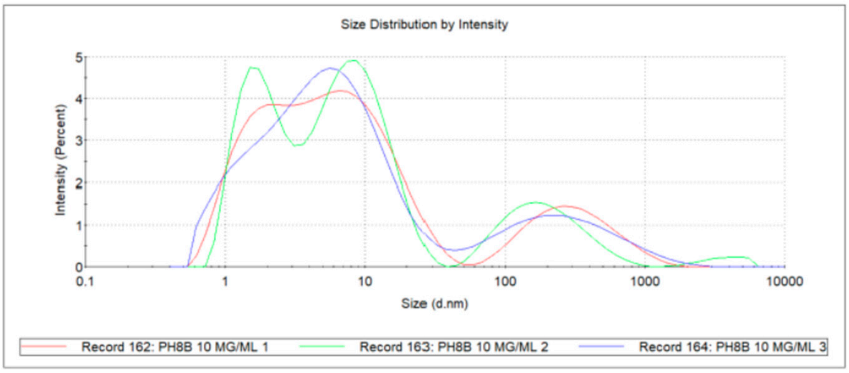

(g1) P9MB 0.1 mg/ml

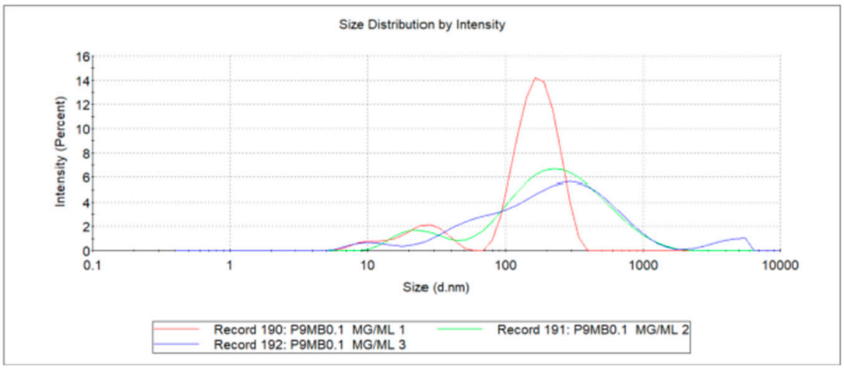

(g2) P9MB 1 mg/ml

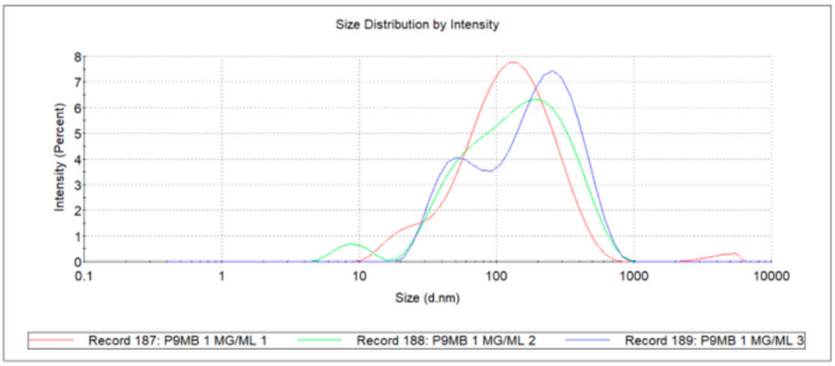

(h1) P10MB 0.1 mg/ml

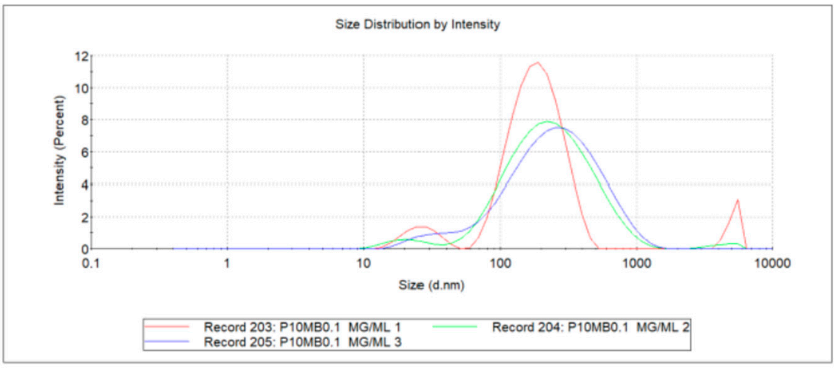

(h2) P10MB 1 mg/ml

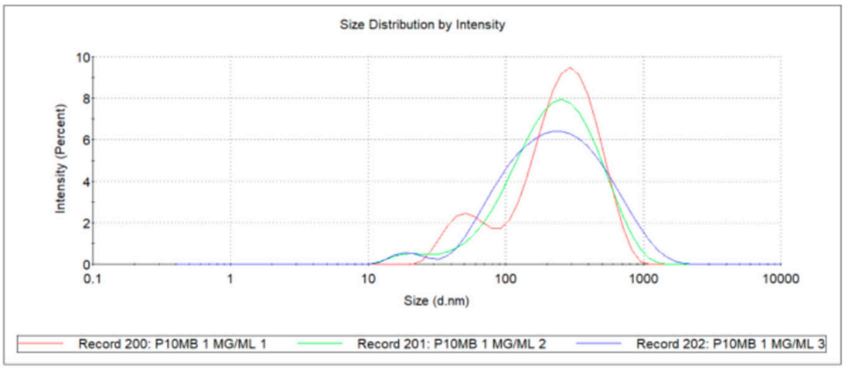

Table S3 *^{1}H NMR chemical shifts for poly(alkylene biguanide) polymers in D_2O and DMSO-d_6.*

Polymer	**–CH₂–, 6–12H**		**4H, –CH₂–**		**4H –CH₂–N–**	
	D_2O	DMSO-d_6	D_2O	DMSO-d_6	D_2O	DMSO-d_6
**P4MB**	-	-	1.54	1.49	3.15	3.11
**P5MB**	1.32	1.32	1.53	1.46	3.14	3.1
**P6MB**	1.29	1.3	1.5	1.45	3.12	3.1
**PHMB**	1.29	1.3	1.49	1.45	3.11	3.09
**P7MB**	1.27	1.28	1.48	1.44	3.13	3.1
**P8MB**	1.25	1.27	1.47	1.44	3.11	3.1
**P9MB**	1.24	1.26	1.48	1.44	3.13	3.09
**P10MB**	1.24	1.25	1.48	1.43	3.12	3.08

Figure S2 *1H NMR spectra of poly(alkyl biguanides) with alkyl chain lengths from C4 to C10 (P4MB, P5MB, P6MB, P7MB, P8MB, P9MB, and P10MB), including marketed poly(hexamethylene biguanide) (PHMB), recorded in DMSO-d_6 at 25 °C.*

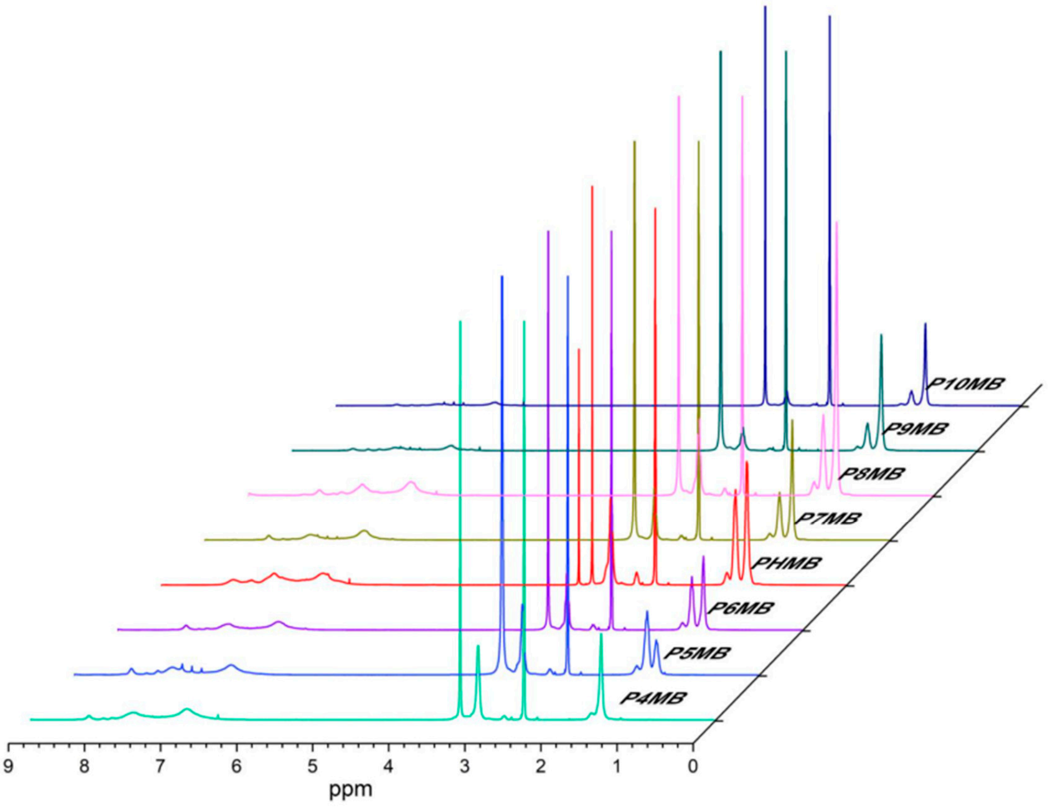

Figure S3 *^{1}H NMR spectra of poly(alkylene biguanide) samples (P4MB–P8mb) in D_2O (400 MHz, 25 °C). Integration values were used for end-group analysis to calculate molecular weight. (a) P4MB, (b) P5MB, (c) P6MB, (d) PHMB, (e) P7MB, (g) P8MB.*

a

P4MB – D_2O

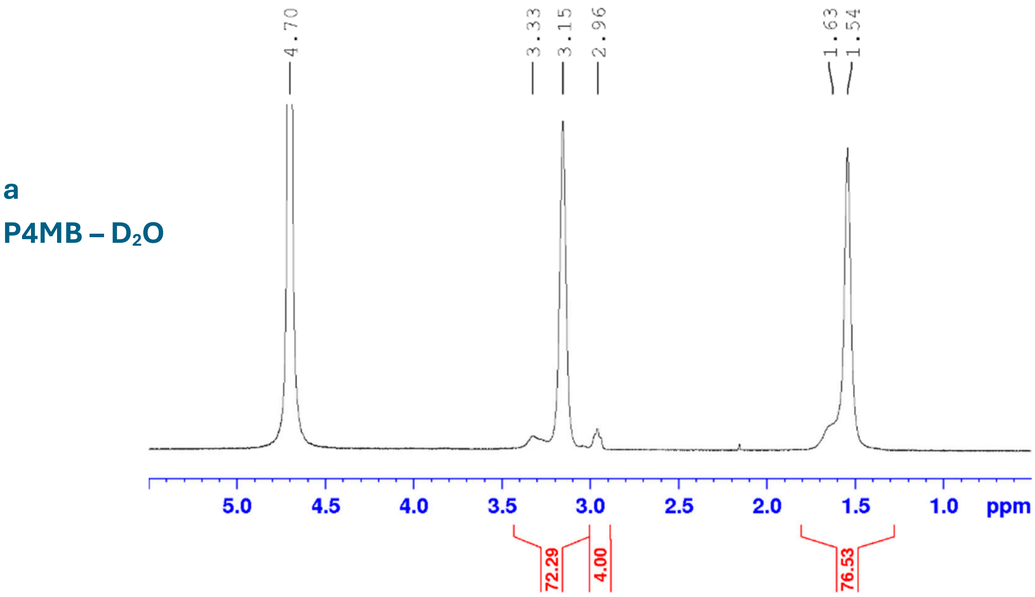

b

P5MB – D_2O

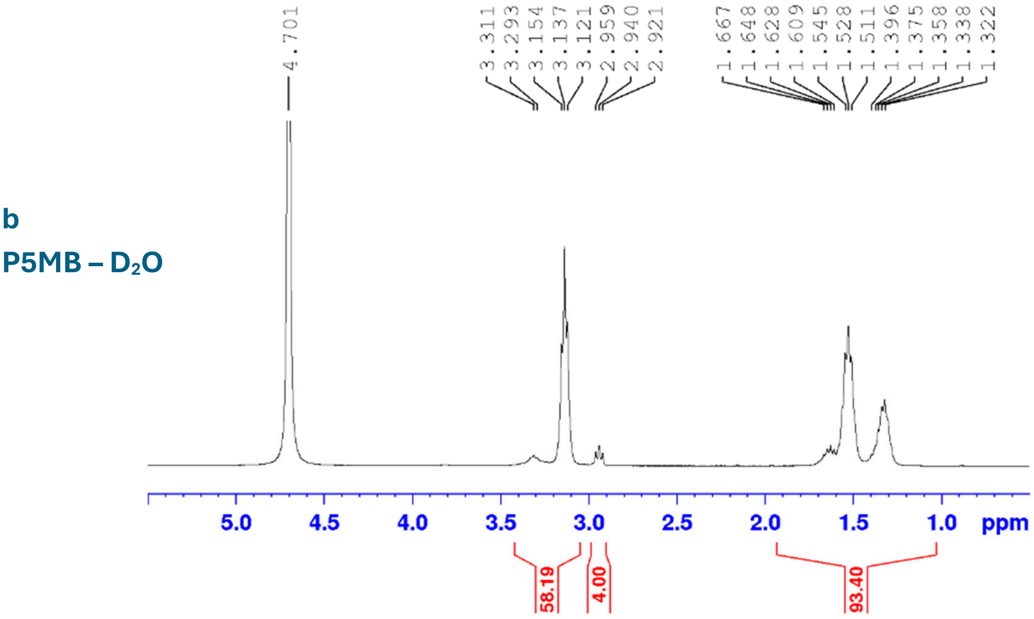

c

P6MB – D_2O

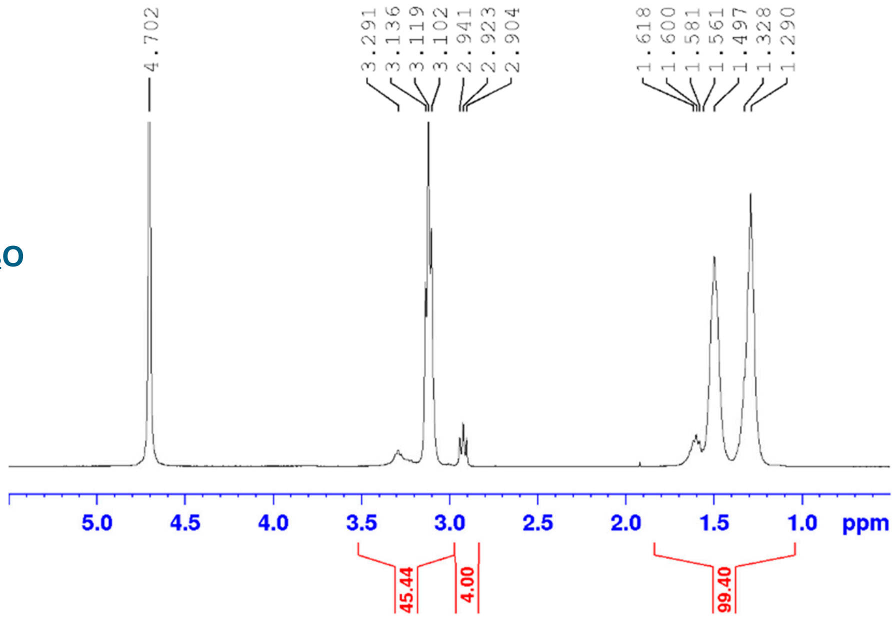

D

PHMB – D_2O

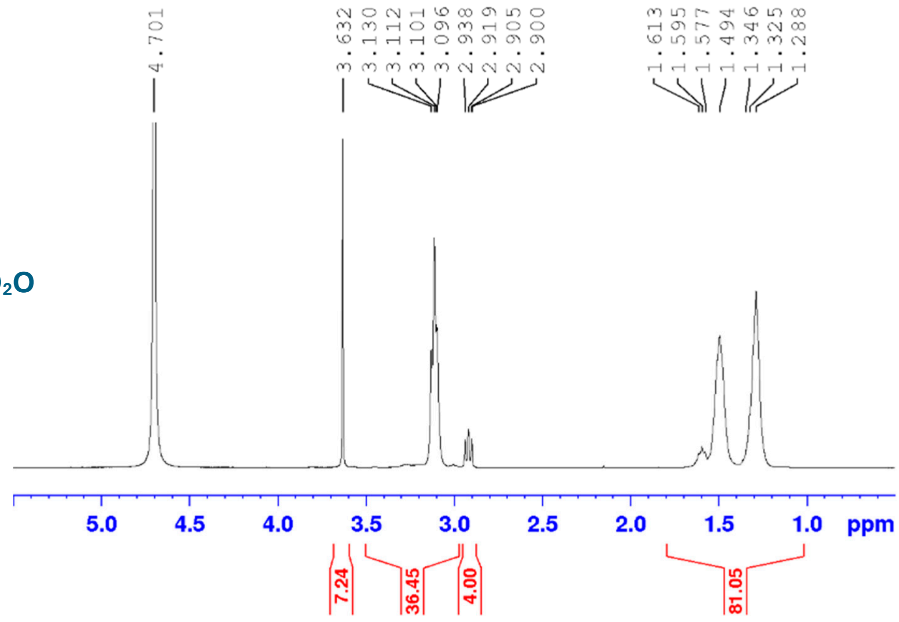

e

P7MB – D_2O

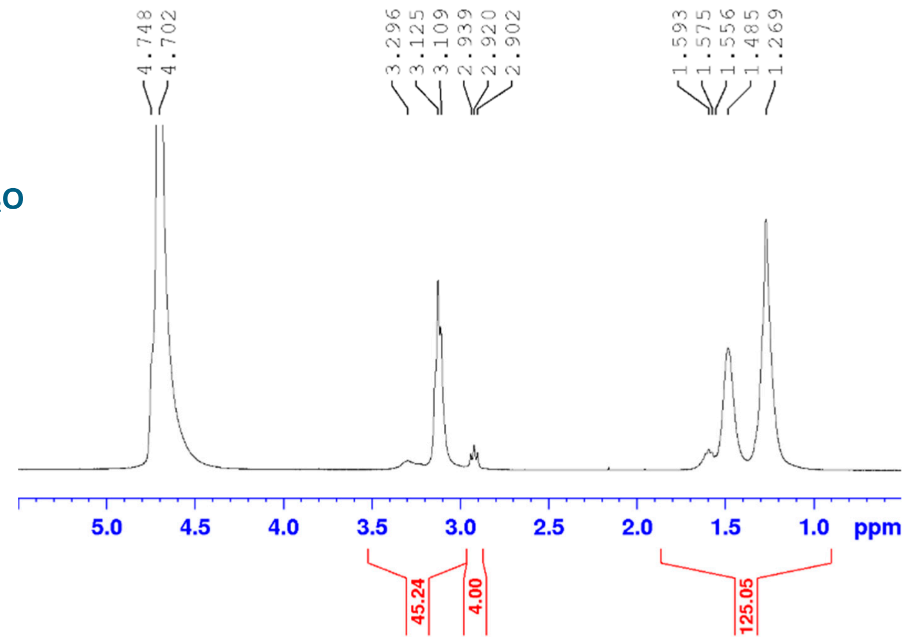

f

P8MB – D_2O

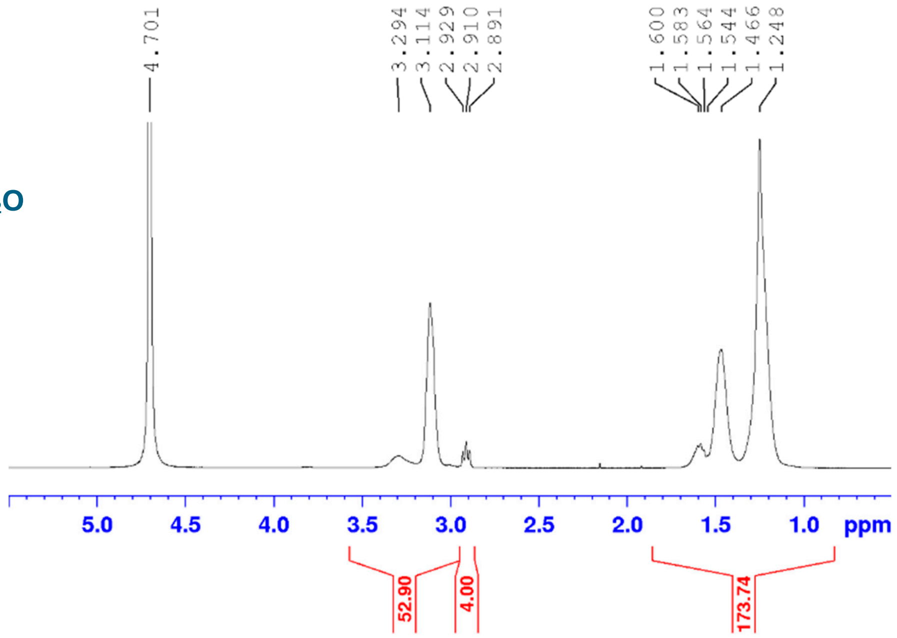

Figure S4 *^{1}H NMR spectra of poly(alkylene biguanide) samples (P4MB–P8MB) in DMSO-d_6 (400 MHz, 25 °C). Integration values were used for end-group analysis to calculate molecular weight. (a) P4MB, (b) P5MB, (c) P6MB, (d) PHMB, (e) P7MB, (f) P8MB, (g) P9MB, (h) P10MB.*

a

P4MB – DMSO-d₆O

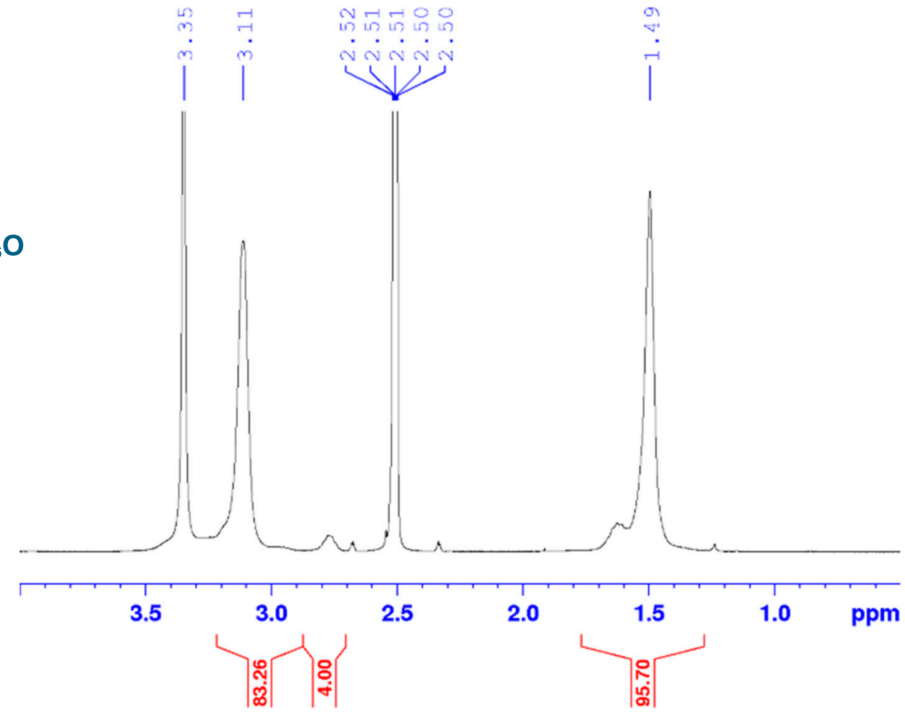

b

P5MB – DMSO-d₆O

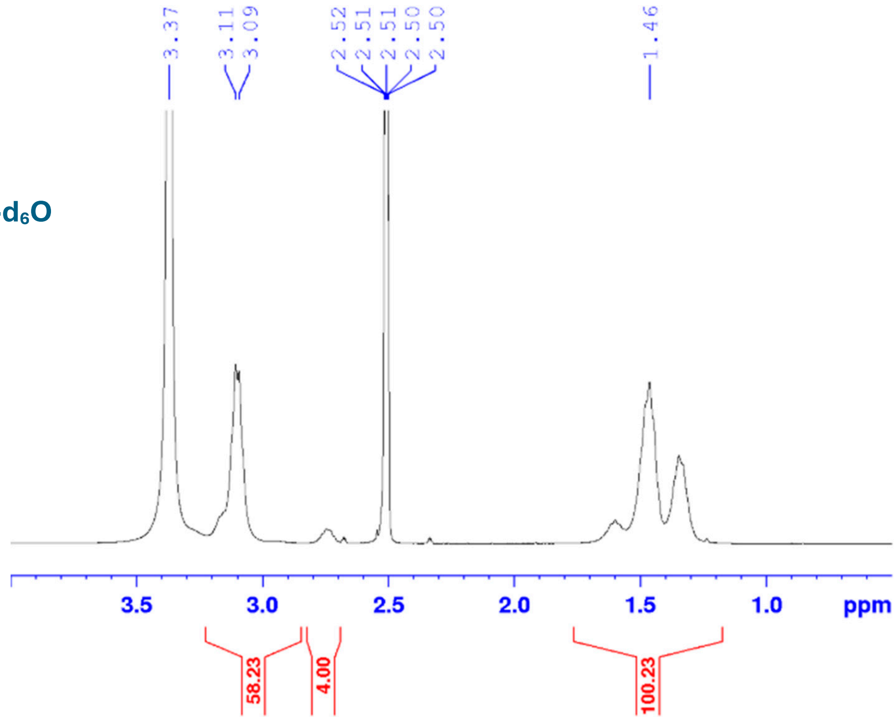

c

P6MB – DMSO-d_6O

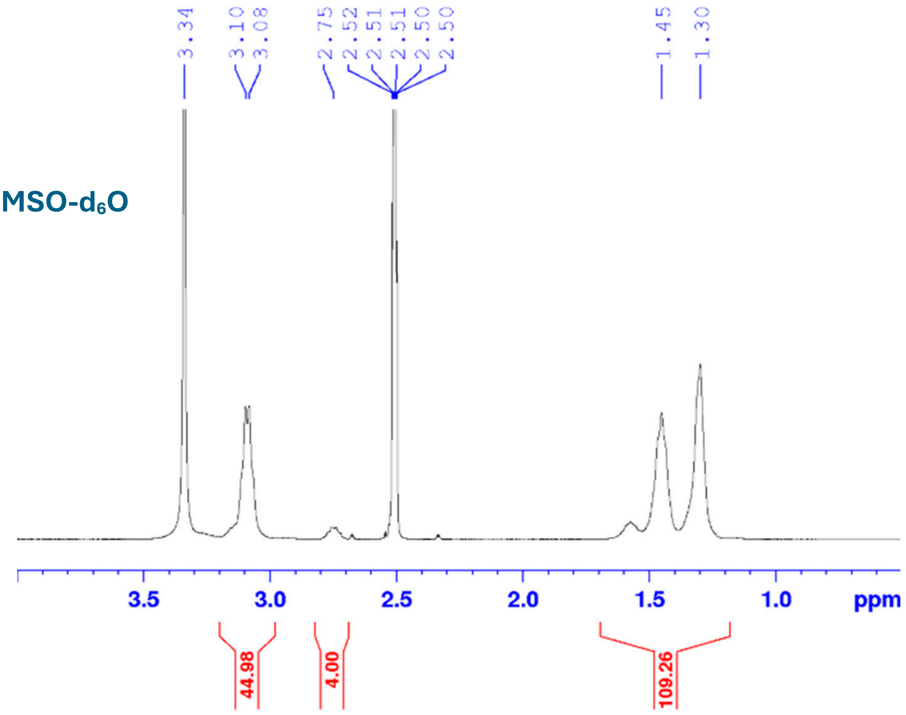

d

PHMB – DMSO-d_6O

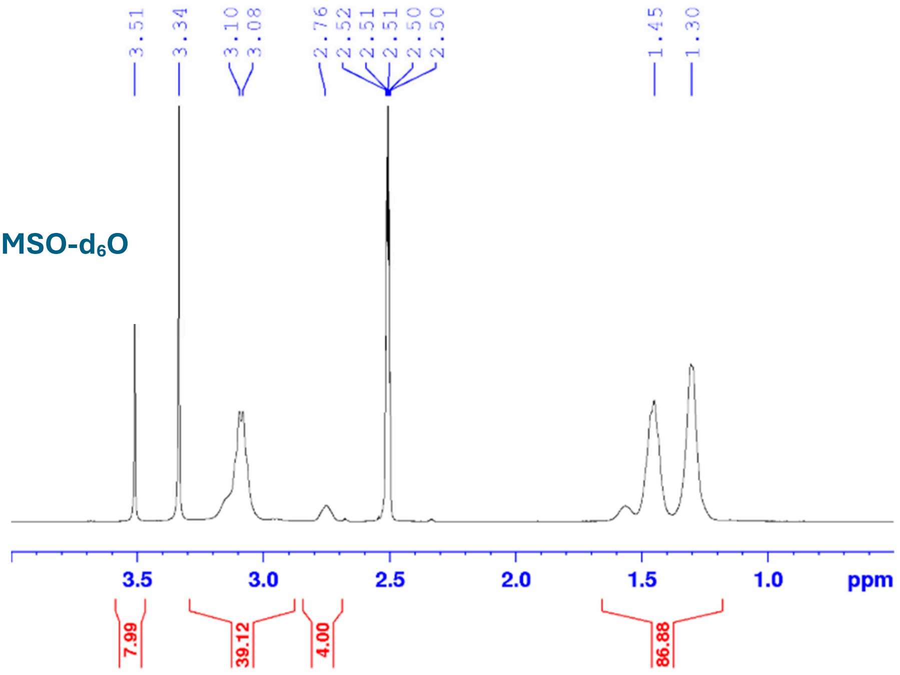

e

P7MB – DMSO-d_6O

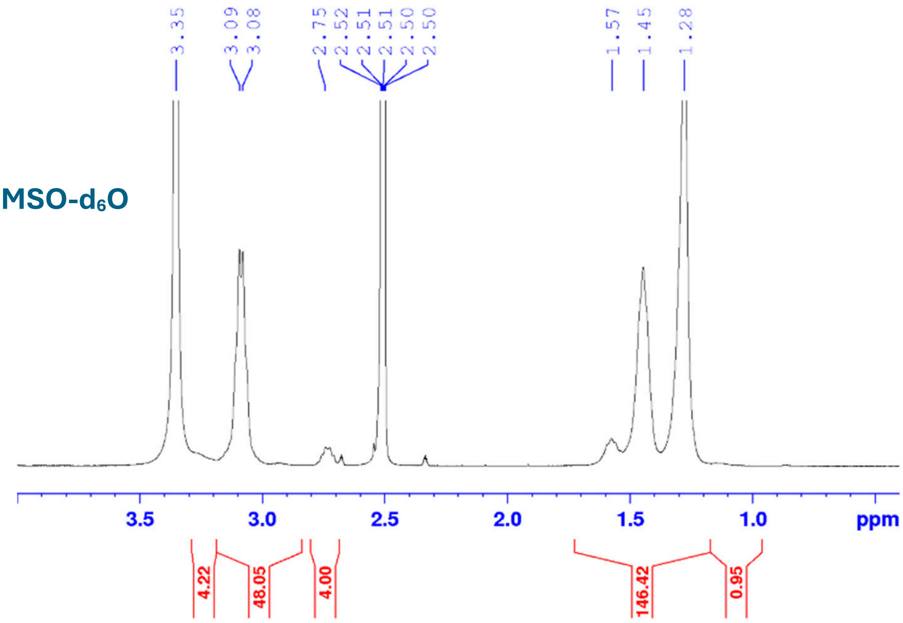

f

P8MB – DMSO-d_6O

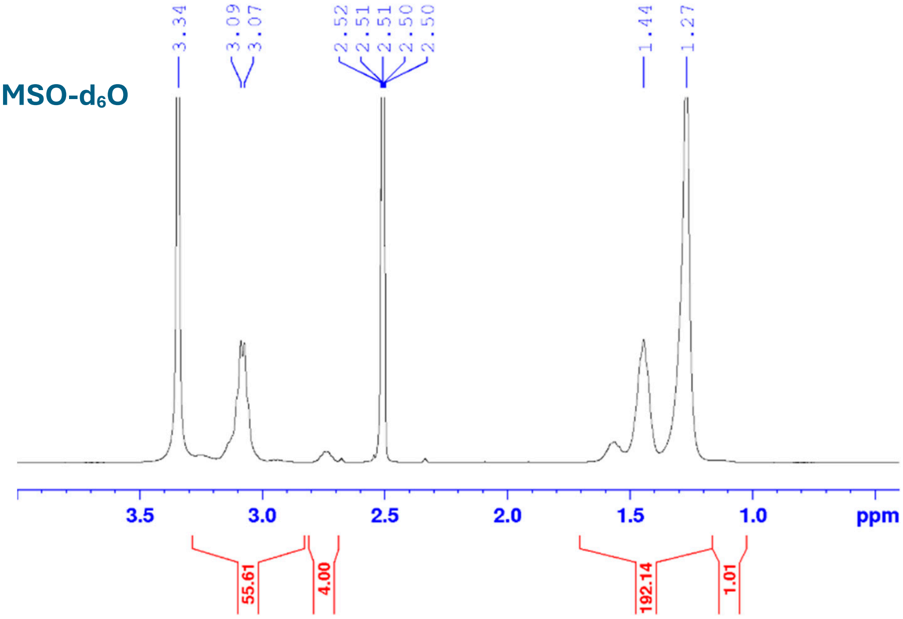

g

P9MB – DMSO-d_6O

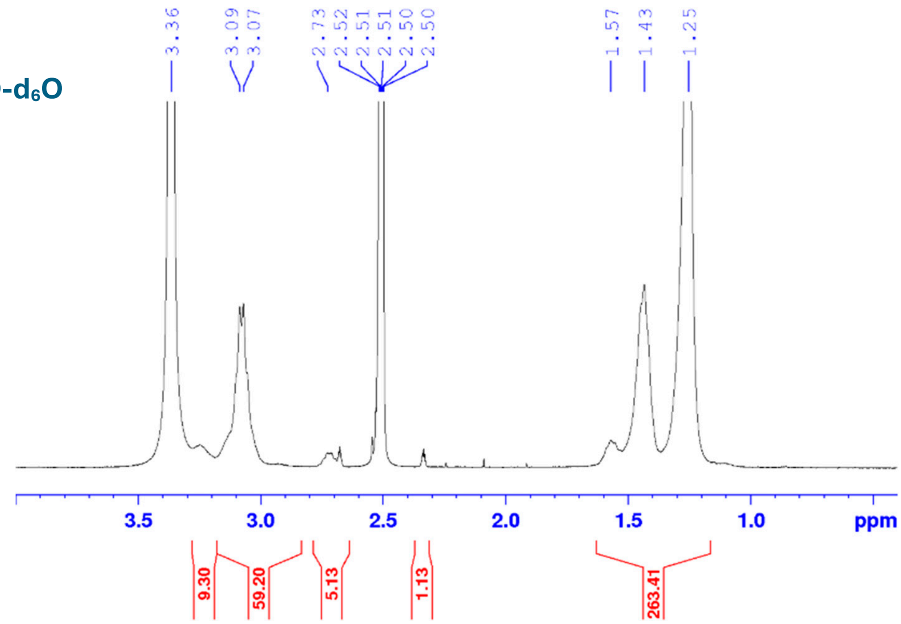

g

P10MB – DMSO-d_6O

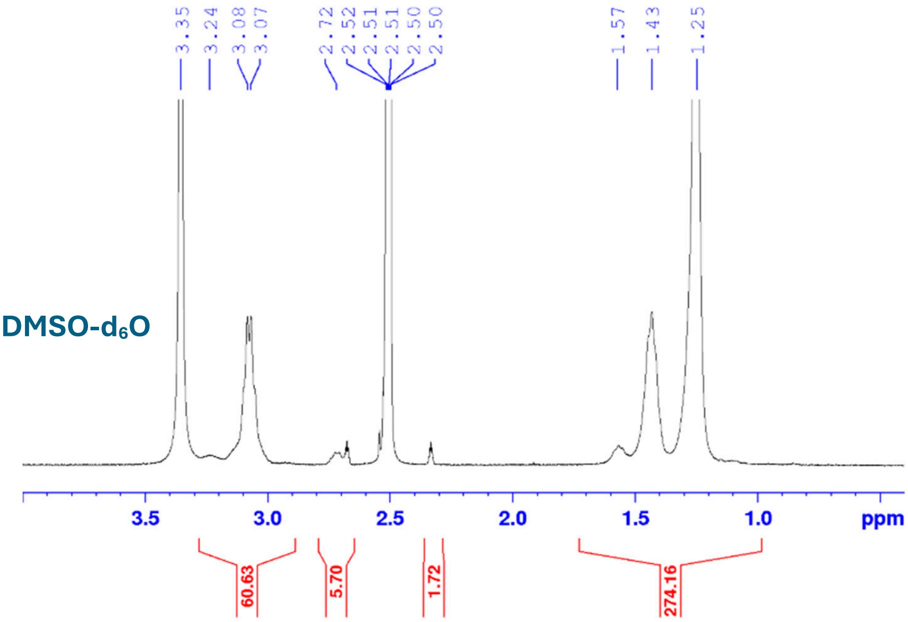

Table S4 *Fractional diffusion coefficient of poly(alkylene biguanide) polymers obtained from 2D DOSY*

**Polymer Type**	**Diffusion Coefficient**			
	**D_2O-Fraction 1**	**D_2O Fraction 2**	**DMSO Fraction 1**	**DMSO Fraction 2**
**P4MB**	1.26 x 10^{-10}	1.53 x 10^{-11}	5.09 x 10^{-11}	-
**P5MB**	1.08 x 10^{-10}	-	4.50 x 10^{-11}	-
**P6MB**	1.14 x 10^{-10}	2.85 x 10^{-11}	5.23 x 10^{-11}	-
**PHMB**	2.01 x 10^{-10}	6.81 x 10^{-11}	7.55 x 10^{-11}	2.71 x 10^{-11}
**P7MB**	1.52 x 10^{-10}	2.80 x 10^{-11}	5.73 x 10^{-11}	-
**P8MB**	1.55 x 10^{-10}	-	5.40 x 10^{-11}	-
**P9MB**	-	-	5.27 x 10^{-11}	1.42 x 10^{-11}
**P10MB**	-	-	4.65 x 10^{-11}	-

Table S5 *2D DOSY spectra and Inverse Laplace Transform figures for poly(alkylene biguanide) polymers in D_2O*

2D DOSY	Inverse Laplace Transform Figures
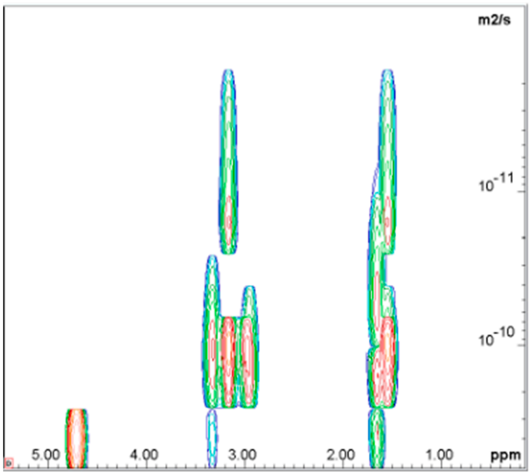	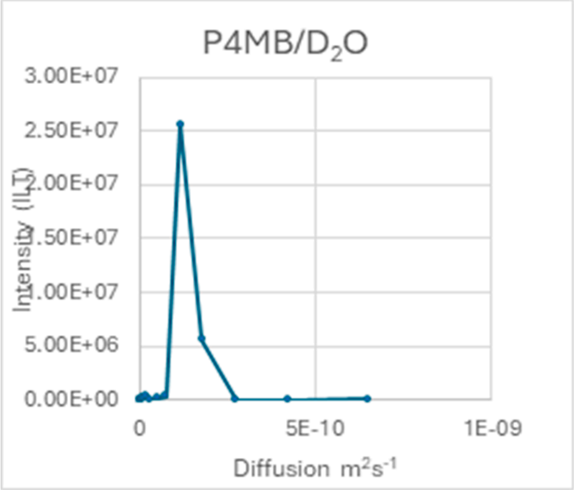
**P4MB in D_2O**	

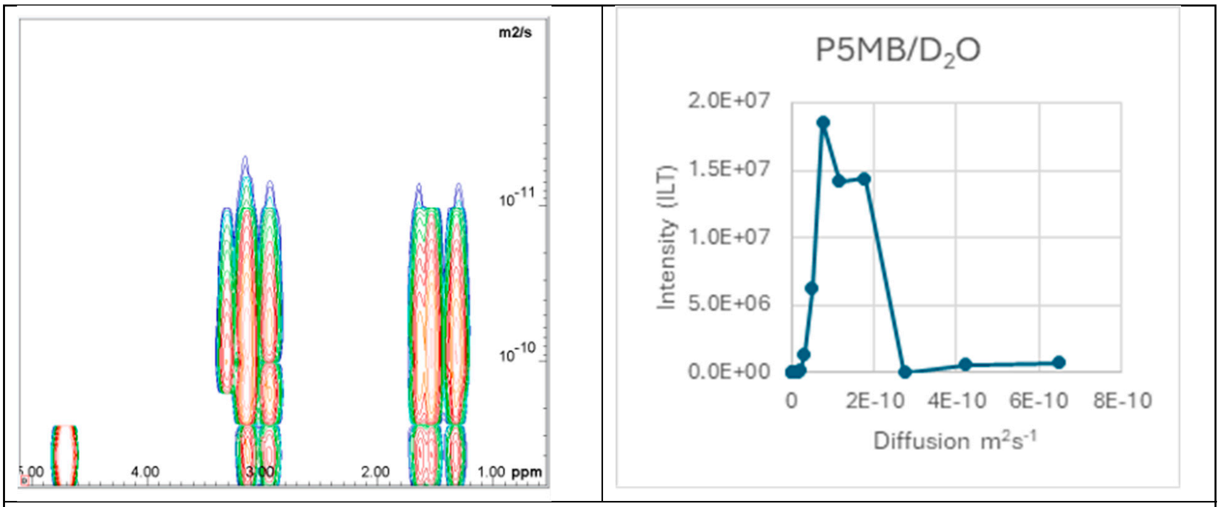

P5MB D_2O

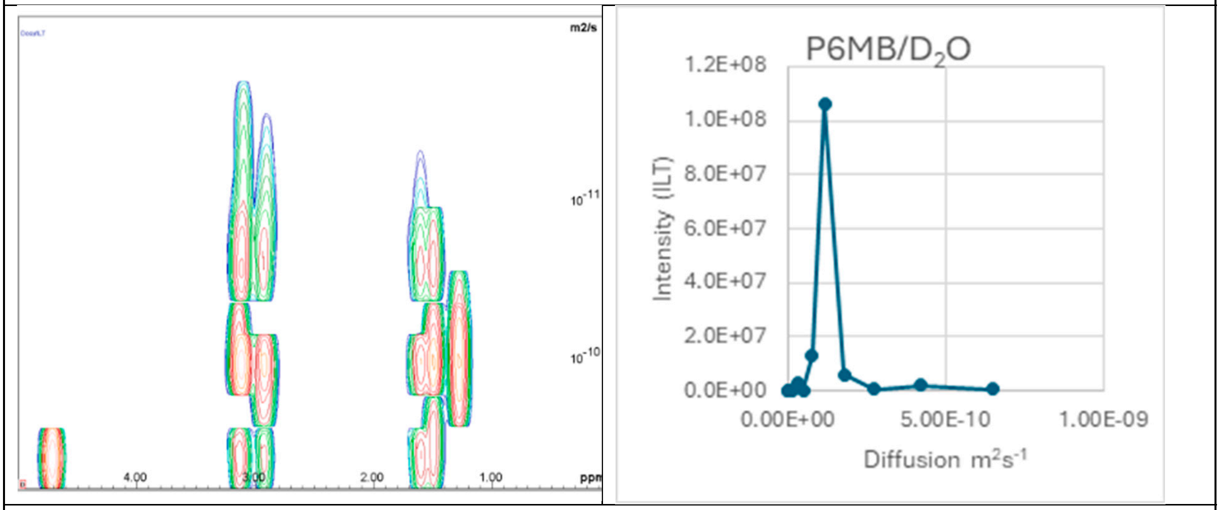

P6MB D_2O

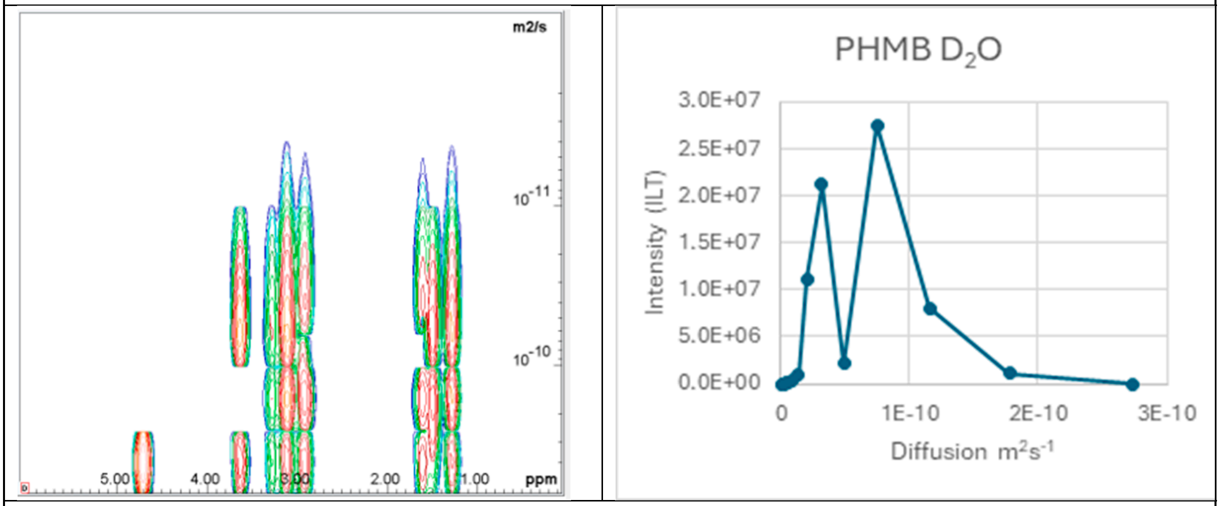

PHMB D_2O

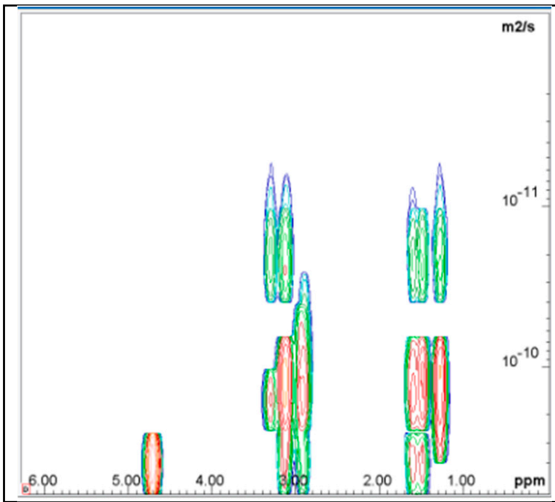

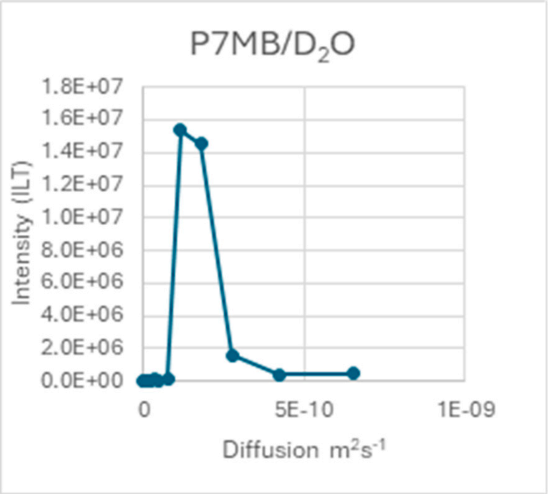

P7MB D_2O

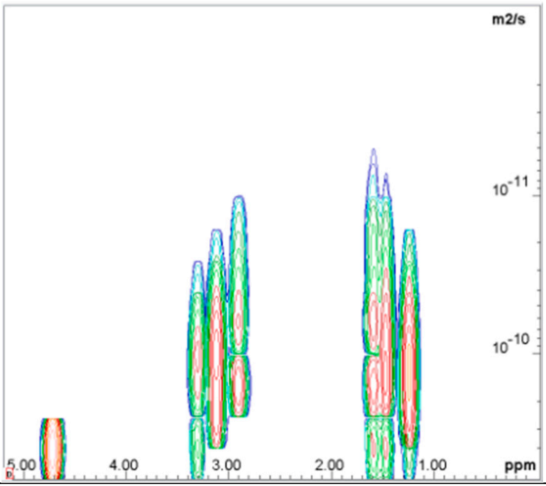

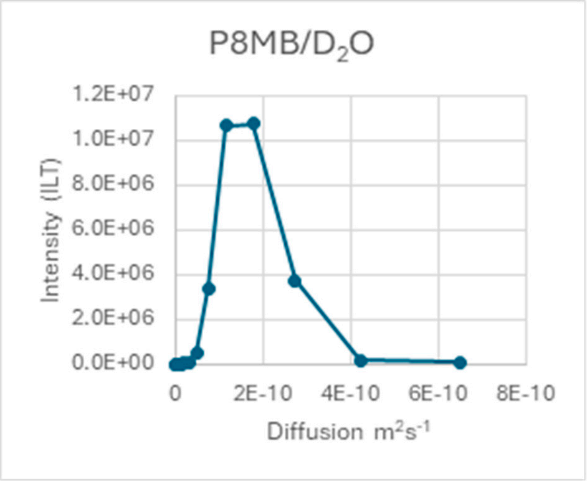

P8MB D_2O

__Table S6__ 2D DOSY spectra and Inverse Laplace Transform figures for poly(alkylene biguanide) polymers in d_6-DMSO

<table>
<tr><th>2D DOSY</th><th>Inverse Laplace Transform Figures</th></tr>
<tr><td>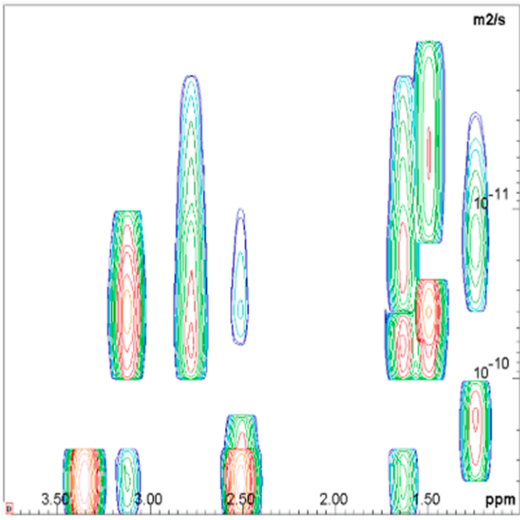
</td><td>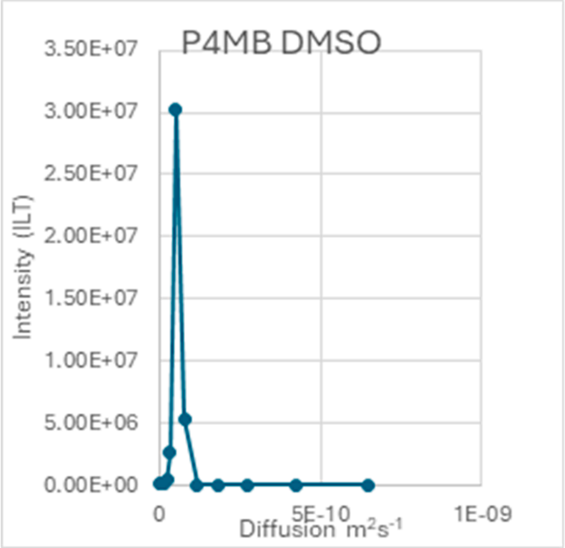
</td></tr>
<tr><td colspan="2">P4MB DMSO-d$_6$</td></tr>
<tr><td>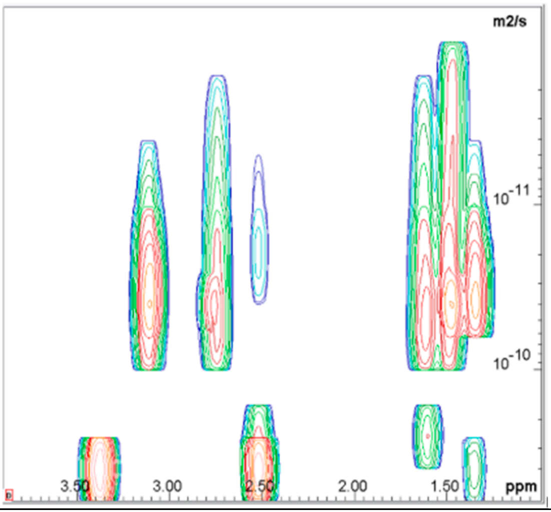
</td><td>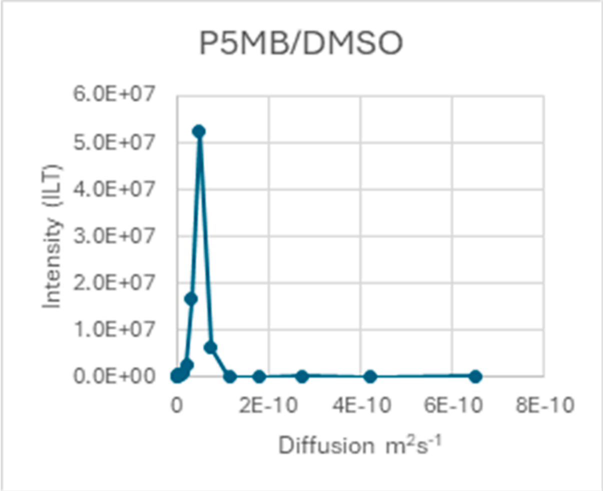
</td></tr>
<tr><td colspan="2">P5MB DMSO-d$_6$</td></tr>
</table>

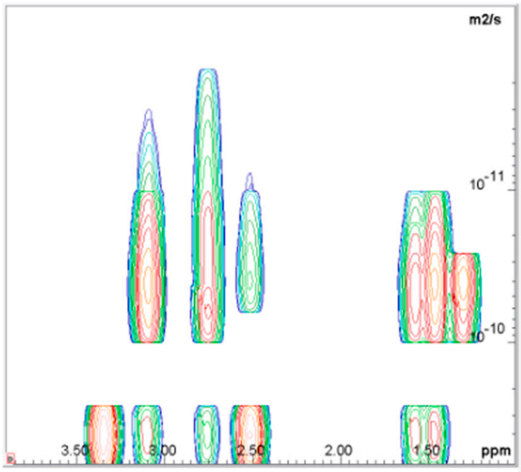

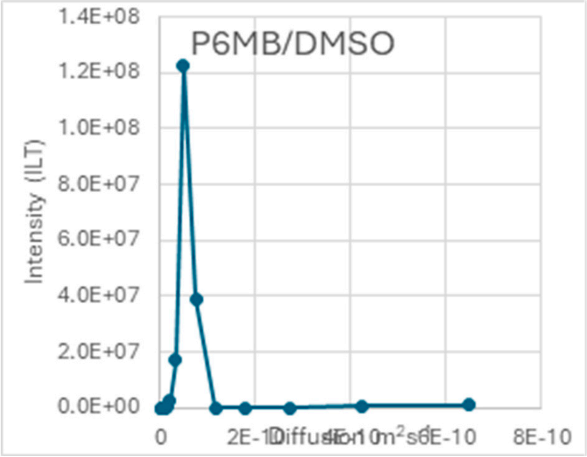

P6MB DMSO-d_6

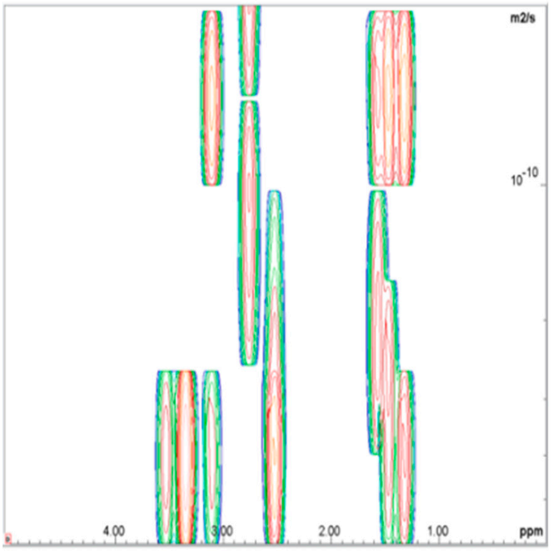

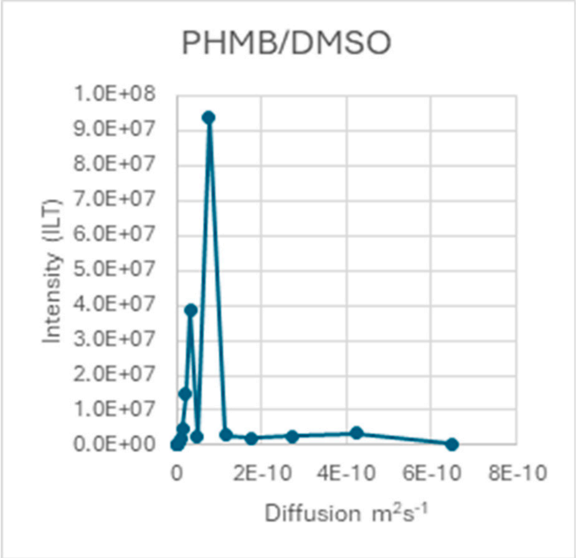

PHMB DMSO-d_6

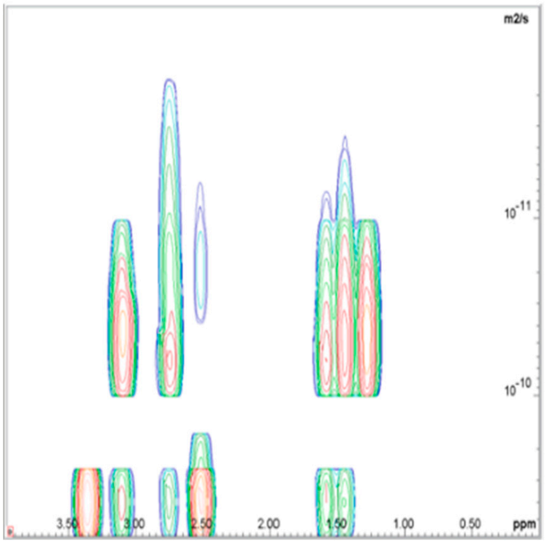

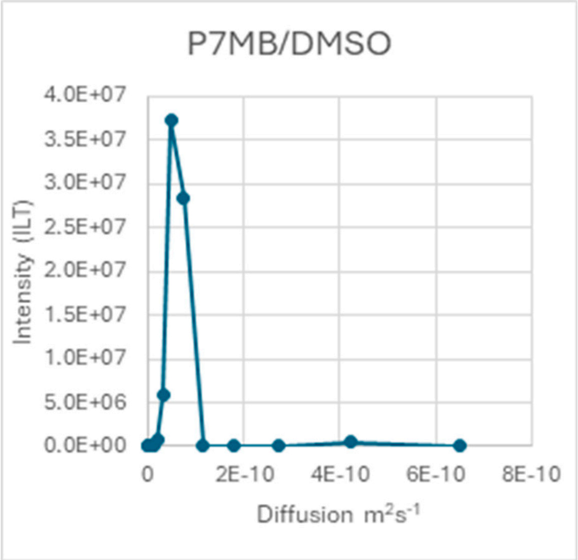

P7MB DMSO-d$_6$

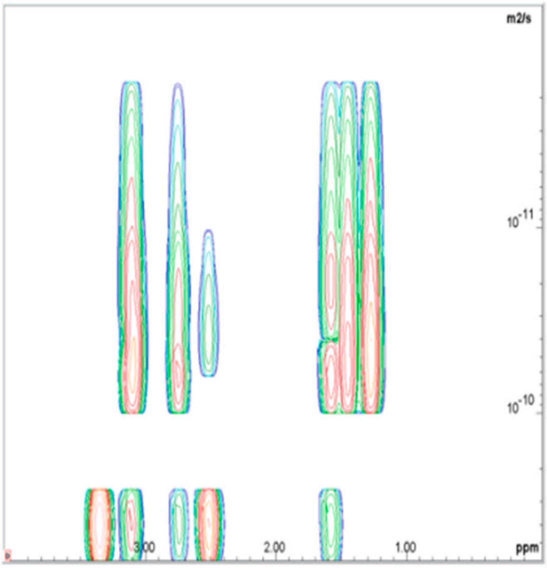

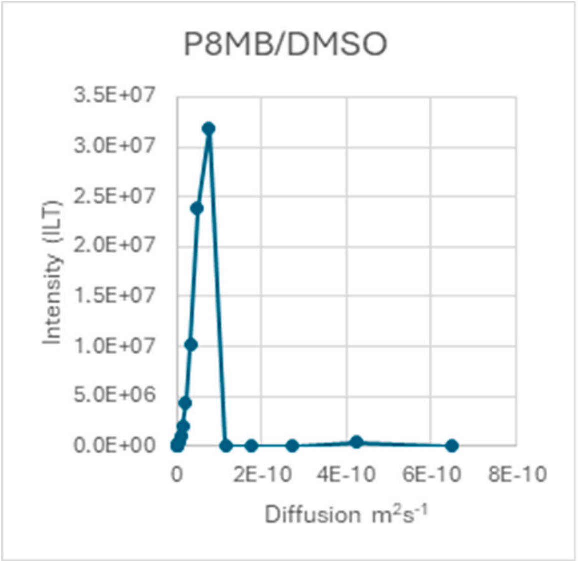

P8MB DMSO-d$_6$

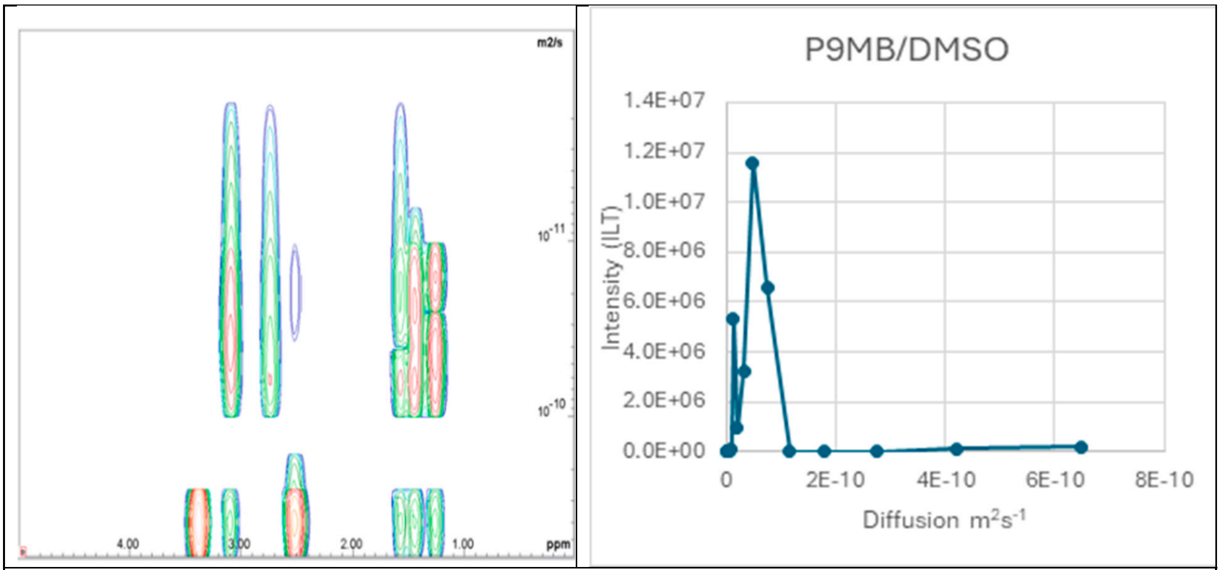

P9MB DMSO-d_6

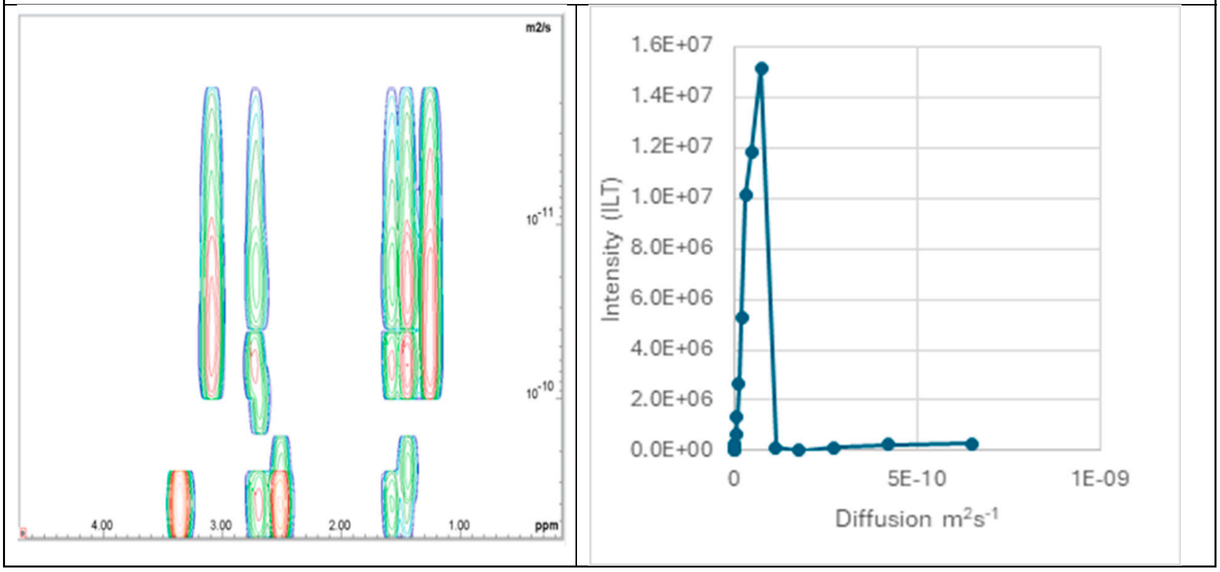

P10MB DMSO-d_6
